# Supplementary material for: Poor sleep and shift work associate with increased blood pressure and inflammation in UK Biobank participants
Source: Nat Commun. 2023 Nov 4;14:7096. doi: 10.1038/s41467-023-42758-6 (PMC10625529; doi:10.1038/s41467-023-42758-6)
Supplement: Supplementary file 1 — Supplementary Information [file 41467_2023_42758_MOESM1_ESM.pdf]

Supplemental Material

Supplementary Table S1. β-estimate ± 95% CI for the association between circadian rhythm-disrupting behaviours and BP.

SLEEP LENGTH AND BP									
		Baseline model		BMI adj model		AH free model		AH free BMI adj model	
	Sleep length	β (95% CI)	p-value	β (95% CI)	p-value	β (95% CI)	p-value	β (95% CI)	p-value
Systolic BP (mmHg)	≤5	1.13 (0.81, 1.46)	6E-12	0.30 (-0.01, 0.62)	0.0619	0.85 (0.52, 1.19)	6E-07	0.18 (-0.15, 0.5)	0.293
	6	0.29 (0.1, 0.47)	2.1E-3	-0.18 (-0.35, 0)	0.0509	0.18 (0, 0.37)	0.0527	-0.21 (-0.39, -0.03)	0.0234
	7	Reference group (β-estimate = 0)							
	8	0.30 (0.14, 0.45)	2.29E-4	0.29 (0.14, 0.44)	0.0002	0.26 (0.1, 0.42)	0.0013	0.27 (0.11, 0.43)	0.0007
	≥9	0.94 (0.66, 1.21)	2E-11	0.48 (0.21, 0.75)	0.0004	0.61 (0.33, 0.9)	3E-05	0.32 (0.04, 0.6)	0.0236
Diastolic BP (mmHg)	≤5	1.03 (0.84, 1.21)	5E-27	0.34 (0.16, 0.51)	0.0002	0.81 (0.62, 1.00)	2E-16	0.22 (0.03, 0.4)	0.0217
	6	0.41 (0.31, 0.52)	1E-14	0.03 (-0.07, 0.13)	0.505	0.31 (0.21, 0.42)	6E-09	-0.02 (-0.12, 0.08)	0.676
	7	Reference group (β-estimate = 0)							
	8	0.29 (0.20, 0.38)	3E-10	0.29 (0.2, 0.37)	8E-11	0.25 (0.15, 0.34)	2E-07	0.25 (0.17, 0.34)	1E-08
	≥9	0.97 (0.81, 1.13)	2E-33	0.59 (0.44, 0.74)	2E-14	0.80 (0.63, 0.96)	3E-21	0.54 (0.38, 0.7)	2E-11
SLEEP QUALITY AND BP									
	Sleep quality score	β (95% CI)	p-value	β	p-value	β (95% CI)	p-value	β (95% CI)	p-value
Systolic BP (mmHg)	4 to 5	Reference group (β-estimate = 0)							
	2 to 3	0.81 (0.64 0.98)	1E-20	0.12 (-0.05, 0.28)	0.169	0.50 (0.33, 0.67)	8E-09	-0.06 (-0.23, 0.10)	0.46
	0 to 1	1.66 (1.41, 1.91)	7E-39	-0.10 (-0.34, 0.15)	0.448	1.03 (0.77, 1.28)	5E-15	-0.44 (-0.69, -0.19)	0.0007
Diastolic BP (mmHg)	4 to 5	Reference group (β-estimate = 0)							
	2 to 3	0.90 (0.80, 1.00)	3E-71	0.33 (0.23, 0.42)	8E-12	0.68 (0.58, 0.78)	8E-42	0.19 (0.10, 0.28)	7E-05
	0 to 1	1.78 (1.63, 1.92)	1E-128	0.33 (0.19, 0.46)	4E-06	1.37 (1.23, 1.52)	2E-73	0.10 (-0.04, 0.24)	0.175
SHIFT WORK AND BP									
	Shift work	β (95% CI)	p-value	β (95% CI)	p-value	β (95% CI)	p-value	β (95% CI)	p-value
Systolic BP (mmHg)	No shift work	Reference group (β-estimate = 0)							
	Day-shift	-0.07 (-0.38, 0.24)	6.54E-1	-0.50 (-0.79, -0.2)	0.001	-0.15 (-0.45, 0.16)	0.339	-0.49 (-0.79, -0.2)	0.0011
	Mixed-shift	0.42 (0.02, 0.81)	3.87E-2	-0.34 (-0.73, 0.04)	0.0773	0.38 (-0.01, 0.77)	0.0557	-0.27 (-0.65, 0.11)	0.169
	Night-shift	-0.17 (-0.87, 0.53)	6.33E-1	-0.91 (-1.59, -0.23)	0.0088	-0.04 (-0.73, 0.65)	0.912	-0.62 (-1.29, 0.06)	0.074

	Permanent night-shift	0.96 (0.38, 1.54)	1.15E-3	0.09 (-0.47, 0.65)	0.754	0.95 (0.38, 1.53)	0.0012	0.20 (-0.36, 0.77)	0.476
**Diastolic BP (mmHg)**	No shift work	Reference group (β-estimate = 0)							
	Day-shift	0.18 (-0.01, 0.36)	5.76E-2	-0.18 (-0.36, -0.01)	0.0395	0.12 (-0.06, 0.3)	0.198	-0.19 (-0.36, -0.01)	0.0368
	Mixed-shift	0.61 (0.37, 0.84)	7E-07	-0.04 (-0.26, 0.18)	0.728	0.51 (0.28, 0.75)	2E-05	-0.06 (-0.28, 0.17)	0.613
	Night-shift	0.30 (-0.12, 0.73)	0.162	-0.31 (-0.71, 0.09)	0.128	0.29 (-0.13, 0.71)	0.171	-0.21 (-0.61, 0.19)	0.3
	Permanent night-shift	0.92 (0.56, 1.27)	3E-07	0.19 (-0.14, 0.52)	0.249	0.89 (0.54, 1.24)	5E-07	0.24 (-0.09, 0.57)	0.152

Supplementary Table S2. β-estimate ± 95% CI for the association between sleep length and BP when stratified by BMI.

RELATIONSHIP BETWEEN SLEEP LENGTH AND BP: STRATIFIED BY BMI								
			**Baseline model**		**AH free model**		**CRP adj model**	
	**BMI CATEGORY**	**Sleep length**	**β**	**p-value**	**β**	**p-value**	**β**	**p-value**
**Systolic BP (mmHg)**	**≤25**	≤5	0.60 (0.01, 1.19)	0.0465	0.36 (-0.23, 0.94)	0.23	0.56 (-0.05, 1.16)	0.0701
		6	-0.03 (-0.34, 0.28)	0.829	-0.05 (-0.35, 0.25)	0.748	-0.04 (-0.35, 0.28)	0.828
		7	Reference group (β-estimate = 0)					
		8	0.40 (0.15, 0.66)	0.0021	0.38 (0.13, 0.63)	0.0027	0.31 (0.04, 0.57)	0.0223
		≥9	0.89 (0.41, 1.36)	0.0002	0.60 (0.12, 1.07)	0.0137	0.77 (0.28, 1.25)	0.002
	**25-<30**	≤5	0.41 (-0.09, 0.90)	0.105	0.42 (-0.09, 0.92)	0.106	0.39 (-0.12, 0.89)	0.133
		6	-0.13 (-0.40, 0.14)	0.36	-0.14 (-0.41, 0.14)	0.329	-0.15 (-0.42, 0.13)	0.297
		7	Reference group (β-estimate = 0)					
		8	0.30 (0.06, 0.53)	0.0131	0.22 (-0.01, 0.46)	0.0662	0.28 (0.04, 0.51)	0.0241
		≥9	0.68 (0.28, 1.09)	0.001	0.47 (0.05, 0.90)	0.0292	0.57 (0.15, 0.98)	0.0074
	**≥30**	≤5	0.30 (-0.29, 0.89)	0.319	-0.10 (-0.75, 0.56)	0.774	0.16 (-0.44, 0.77)	0.597
		6	-0.26 (-0.63, 0.11)	0.167	-0.42 (-0.82, -0.02)	0.0384	-0.37 (-0.75, 0.002)	0.0512
		7	Reference group (β-estimate = 0)					
		8	0.05 (-0.29, 0.39)	0.762	0.12 (-0.25, 0.50)	0.517	-0.01 (-0.36, 0.34)	0.972
		≥9	0.02 (-0.51, 0.56)	0.935	-0.19 (-0.81, 0.43)	0.552	-0.11 (-0.65, 0.44)	0.707
**Diastolic BP (mmHg)**	**≤25**	≤5	0.42 (0.09, 0.75)	0.0117	0.28 (-0.05, 0.61)	0.093	0.42 (0.08, 0.76)	0.015
		6	0.16 (-0.01, 0.33)	0.0732	0.13 (-0.04, 0.30)	0.146	0.14 (-0.04, 0.32)	0.117
		7	Reference group (β-estimate = 0)					
		8	0.28 (0.14, 0.42)	0.0001	0.27 (0.13, 0.41)	0.0001	0.24 (0.10, 0.39)	0.0011
		≥9	0.80 (0.53, 1.06)	4E-09	0.66 (0.40, 0.93)	1E-06	0.69 (0.41, 0.96)	8E-07
	**25-<30**	≤5	0.42 (0.14, 0.70)	0.003	0.33 (0.04, 0.61)	0.0251	0.39 (0.11, 0.68)	0.0067
		6	-0.001 (-0.15, 0.15)	0.985	-0.04 (-0.19, 0.12)	0.631	-0.02 (-0.18, 0.13)	0.786
		7	Reference group (β-estimate = 0)					
		8	0.27 (0.13, 0.40)	8E-05	0.20 (0.07, 0.33)	0.0033	0.26 (0.12, 0.39)	0.0002
		≥9	0.77 (0.54, 1.00)	6E-11	0.70 (0.46, 0.94)	1E-08	0.67 (0.43, 0.90)	2E-08
	**≥30**	≤5	0.50 (0.16, 0.83)	0.0036	0.25 (-0.12, 0.62)	0.185	0.42 (0.08, 0.77)	0.0161

		6	0.09 (-0.12, 0.30)	0.404	-0.06 (-0.28, 0.17)	0.622	0.05 (-0.16, 0.26)	0.634
		7	Reference group (β-estimate = 0)					
		8	0.35 (0.15, 0.54)	0.0005	0.34 (0.12, 0.55)	0.0019	0.32 (0.12, 0.51)	0.0019
		≥9	0.40 (0.09, 0.70)	0.0101	0.27 (-0.08, 0.63)	0.127	0.30 (-0.01, 0.62)	0.0556

Supplementary Table S3. β-estimate ± 95% CI for the association between sleep length and BP when stratified by BMI and sex.

SLEEP LENGTH AND BP: STRATIFIED BY BMI AND SEX				
		Baseline model		
	**BMI AND SEX CATEGORY**	**Sleep length**	**β (95% CI)**	**p-value**
**Systolic BP (mmHg)**	**Males ≤25**	≤5	0.53 (-0.21, 1.27)	0.158
		6	-0.05 (-0.45, 0.34)	0.793
		7	Reference group (β-estimate = 0)	
		8	0.50 (0.18, 0.82)	0.0024
		≥9	0.87 (0.27, 1.47)	0.0045
	**Males >25**	≤5	0.90 (0.33, 1.46)	0.0019
		6	0.03 (-0.32, 0.37)	0.88
		7	Reference group (β-estimate = 0)	
		8	0.01 (-0.29, 0.30)	0.957
		≥9	0.71 (0.22, 1.19)	0.0044
	**Females ≤25**	≤5	0.32 (-0.65, 1.29)	0.517
		6	-0.26 (-0.76, 0.23)	0.296
		7	Reference group (β-estimate = 0)	
		8	0.64 (0.22, 1.07)	0.0029
		≥9	1.64 (0.86, 2.41)	4E-05
	**Females >25**	≤5	0.49 (-0.03, 1.00)	0.0626
		6	-0.07 (-0.35, 0.21)	0.632
		7	Reference group (β-estimate = 0)	
		8	0.63 (0.37, 0.89)	2E-06
		≥9	0.95 (0.51, 1.38)	2E-05
**Diastolic BP (mmHg)**	**Males ≤25**	≤5	0.36 (-0.05, 0.76)	0.083
		6	0.11 (-0.10, 0.33)	0.306
		7	Reference group (β-estimate = 0)	
		8	0.25 (0.07, 0.42)	0.006
		≥9	0.76 (0.43, 1.09)	6E-06
	**Males >25**	≤5	0.90 (0.59, 1.22)	2E-08
		6	0.25 (0.06, 0.44)	0.01
		7	Reference group (β-estimate = 0)	
		8	0.16 (-0.01, 0.32)	0.0597
		≥9	0.73 (0.46, 1.00)	1E-07
	**Females ≤25**	≤5	0.48 (-0.09, 1.04)	0.0984
		6	0.19 (-0.09, 0.48)	0.19
		7	Reference group (β-estimate = 0)	
		8	0.40 (0.15, 0.64)	0.0016
		≥9	0.94 (0.49, 1.39)	4E-05
	**Females >25**	≤5	0.68 (0.38, 0.98)	1E-05
		6	0.17 (0, 0.33)	0.0507
		7	Reference group (β-estimate = 0)	
		8	0.45 (0.29, 0.60)	9E-09
		≥9	0.91 (0.65, 1.17)	8E-12

Supplementary Table S4A. β-estimate ± 95% CI for the association between sleep length and BP when stratified by sex and age.

SLEEP LENGTH AND BP: STRATIFIED BY SEX										
			**Baseline model**		**BMI adj model**		**AH free model**		**AH free BMI adj model**	
	**SEX**	**Sleep length**	**β (95% CI)**	**p-value**	**β (95% CI)**	**p-value**	**β (95% CI)**	**p-value**	**β (95% CI)**	**p-value**
**Systolic BP (mmHg)**	**Males**	≤5	0.81 (0.35, 1.26)	0.0006	0.03 (-0.42, 0.48)	0.889	0.56 (0.08, 1.04)	0.0229	-0.08 (-0.55, 0.40)	0.756
		6	0.14 (-0.11, 0.39)	0.275	-0.36 (-0.60, -0.11)	0.0043	-0.03 (-0.28, 0.22)	0.814	-0.43 (-0.68, -0.18)	0.0007
		7	Reference group (β-estimate = 0)							
		8	0.66 (0.43, 0.88)	8E-09	0.63 (0.41, 0.85)	2E-08	0.57 (0.34, 0.80)	1E-06	0.57 (0.34, 0.79)	1E-06
		≥9	1.28 (0.89, 1.67)	8E-11	0.80 (0.42, 1.18)	3E-05	0.88 (0.46, 1.30)	4E-05	0.57 (0.15, 0.98)	0.0072
	**Females**	≤5	1.22 (0.76, 1.67)	2E-07	0.36 (-0.08, 0.80)	0.113	0.86 (0.40, 1.32)	0.0003	0.18 (-0.27, 0.63)	0.433
		6	0.26 (-0.01, 0.52)	0.0565	-0.16 (-0.42, 0.10)	0.217	0.17 (-0.09, 0.44)	0.194	-0.19 (-0.45, 0.07)	0.15
		7	Reference group (β-estimate = 0)							
		8	0.26 (0.04, 0.49)	0.0192	0.24 (0.03, 0.46)	0.0284	0.34 (0.12, 0.56)	0.0026	0.32 (0.11, 0.54)	0.0031
		≥9	1.11 (0.73, 1.50)	1E-08	0.62 (0.24, 0.99)	0.0012	0.91 (0.53, 1.30)	4E-06	0.59 (0.21, 0.97)	0.0022
**Diastolic BP (mmHg)**	**Males**	≤5	0.93 (0.66, 1.20)	3E-11	0.29 (0.03, 0.56)	0.0287	0.74 (0.45, 1.02)	6E-07	0.18 (-0.10, 0.45)	0.215
		6	0.38 (0.23, 0.53)	5E-07	-0.01 (-0.16, 0.13)	0.847	0.28 (0.13, 0.43)	0.0003	-0.07 (-0.22, 0.08)	0.36
		7	Reference group (β-estimate = 0)							
		8	0.45 (0.32, 0.59)	2E-11	0.43 (0.30, 0.56)	4E-11	0.36 (0.22, 0.50)	3E-07	0.36 (0.23, 0.49)	1E-07
		≥9	1.06 (0.83, 1.29)	2E-19	0.65 (0.43, 0.87)	7E-09	0.85 (0.59, 1.10)	6E-11	0.57 (0.32, 0.81)	5E-06
	**Females**	≤5	1.06 (0.80, 1.32)	5E-16	0.34 (0.09, 0.58)	0.0064	0.81 (0.55, 1.07)	1E-09	0.20 (-0.05, 0.45)	0.11
		6	0.41 (0.26, 0.55)	8E-08	0.05 (-0.09, 0.19)	0.483	0.30 (0.15, 0.44)	8E-05	-0.02 (-0.16, 0.12)	0.777
		7	Reference group (β-estimate = 0)							
		8	0.23 (0.10, 0.35)	0.0004	0.21 (0.09, 0.32)	0.0006	0.24 (0.12, 0.37)	0.0001	0.23 (0.11, 0.34)	0.0001
		≥9	1.02 (0.80, 1.23)	3E-20	0.60 (0.39, 0.80)	9E-09	0.90 (0.68, 1.12)	7E-16	0.61 (0.40, 0.81)	8E-09
**SLEEP LENGTH AND BP: STRATIFIED BY AGE**										
	**AGE**	**Sleep length**	**β (95% CI)**	**p-value**	**β (95% CI)**	**p-value**	**β (95% CI)**	**p-value**	**β (95% CI)**	**p-value**
**Systolic BP (mmHg)**	**≤50**	≤5	1.32 (0.73, 1.90)	9E-06	0.09 (-0.47, 0.65)	0.747	1.29 (0.72, 1.85)	9E-06	0.26 (-0.29, 0.81)	0.351
		6	0.46 (0.16, 0.76)	0.0027	-0.22 (-0.51, 0.07)	0.136	0.37 (0.08, 0.66)	0.0137	-0.23 (-0.51, 0.05)	0.109
		7	Reference group (β-estimate = 0)							
		8	0.08 (-0.18, 0.34)	0.563	0.23 (-0.02, 0.48)	0.0692	0.05 (-0.21, 0.30)	0.723	0.18 (-0.06, 0.42)	0.144

		≥9	0.67 (0.14, 1.20)	0.0136	0.39 (-0.12, 0.90)	0.133	0.49 (-0.02, 1.01)	0.0617	0.27 (-0.22, 0.77)	0.282
	**>50**	≤5	1.12 (0.73, 1.51)	2E-08	0.41 (0.03, 0.80)	0.0356	0.69 (0.28, 1.11)	0.0011	0.16 (-0.25, 0.57)	0.451
		6	0.22 (-0.01, 0.44)	0.0588	-0.15 (-0.37, 0.07)	0.173	0.07 (-0.16, 0.31)	0.543	-0.22 (-0.45, 0.01)	0.0654
		7	Reference group (β-estimate = 0)							
		8	0.50 (0.30, 0.69)	6E-07	0.43 (0.24, 0.62)	1E-05	0.49 (0.29, 0.70)	2E-06	0.45 (0.25, 0.65)	1E-05
		≥9	1.32 (1.00, 1.65)	1E-15	0.82 (0.50, 1.14)	4E-07	0.98 (0.63, 1.33)	4E-08	0.67 (0.32, 1.01)	0.0001
**Diastolic BP (mmHg)**	**≤50**	≤5	1.42 (1.04, 1.81)	4E-13	0.35 (-0.01, 0.71)	0.0538	1.31 (0.94, 1.69)	9E-12	0.38 (0.02, 0.73)	0.0372
		6	0.63 (0.43, 0.83)	5E-10	0.03 (-0.15, 0.22)	0.748	0.53 (0.33, 0.72)	9E-08	-0.02 (-0.20, 0.16)	0.845
		7	Reference group (β-estimate = 0)							
		8	0.14 (-0.04, 0.31)	0.121	0.26 (0.11, 0.42)	0.0011	0.11 (-0.06, 0.27)	0.204	0.22 (0.07, 0.38)	0.0047
		≥9	0.97 (0.62, 1.32)	6E-08	0.73 (0.40, 1.06)	1E-05	0.88 (0.53, 1.22)	5E-07	0.68 (0.36, 1.00)	3E-05
	**>50**	≤5	0.87 (0.66, 1.09)	1E-15	0.30 (0.10, 0.51)	0.0038	0.58 (0.36, 0.81)	4E-07	0.12 (-0.10, 0.33)	0.29
		6	0.30 (0.17, 0.42)	3E-06	-0.001 (-0.12, 0.12)	0.991	0.18 (0.05, 0.30)	0.0067	-0.07 (-0.19, 0.05)	0.271
		7	Reference group (β-estimate = 0)							
		8	0.38 (0.27, 0.48)	8E-12	0.32 (0.22, 0.42)	1E-09	0.34 (0.23, 0.45)	1E-09	0.30 (0.20, 0.41)	3E-08
		≥9	1.04 (0.86, 1.21)	2E-30	0.62 (0.45, 0.79)	8E-13	0.85 (0.66, 1.04)	1E-18	0.57 (0.39, 0.75)	9E-10

Supplementary Table S4B. β-estimate ± 95% CI for the association between sleep length and BP when stratified by sex and age.

SLEEP LENGTH AND BP: STRATIFIED BY SEX						
			**CRP adj model**		**BMI and CRP adj model**	
	**SEX**	**Sleep length**	**β (95% CI)**	**p-value**	**β (95% CI)**	**p-value**
**Systolic BP (mmHg)**	**Males**	≤5	0.59 (0.13, 1.06)	0.0128	0.03 (-0.43, 0.49)	0.903
		6	0.01 (-0.24, 0.26)	0.95	-0.39 (-0.64, -0.14)	0.0019
		7	Reference group (β-estimate = 0)			
		8	0.61 (0.38, 0.84)	2E-07	0.62 (0.39, 0.84)	7E-08
		≥9	1.08 (0.68, 1.47)	9E-08	0.79 (0.40, 1.18)	6E-05
	**Females**	≤5	0.87 (0.41, 1.33)	0.0002	0.32 (-0.13, 0.78)	0.165
		6	0.11 (-0.16, 0.37)	0.44	-0.17 (-0.44, 0.09)	0.192
		7	Reference group (β-estimate = 0)			
		8	0.11 (-0.12, 0.33)	0.346	0.16 (-0.06, 0.38)	0.151
		≥9	0.68 (0.29, 1.06)	0.0006	0.46 (0.07, 0.84)	0.0192
**Diastolic BP (mmHg)**	**Males**	≤5	0.76 (0.48, 1.04)	8E-08	0.28 (0.01, 0.55)	0.0413
		6	0.29 (0.14, 0.44)	0.0002	-0.04 (-0.18, 0.11)	0.63
		7	Reference group (β-estimate = 0)			
		8	0.41 (0.28, 0.55)	2E-09	0.42 (0.29, 0.55)	3E-10
		≥9	0.84 (0.61, 1.08)	2E-12	0.59 (0.37, 0.82)	3E-07
	**Females**	≤5	0.82 (0.56, 1.08)	5E-10	0.33 (0.08, 0.57)	0.01
		6	0.29 (0.14, 0.44)	0.0001	0.04 (-0.11, 0.18)	0.605
		7	Reference group (β-estimate = 0)			
		8	0.14 (0.01, 0.26)	0.0344	0.18 (0.06, 0.30)	0.0032
		≥9	0.70 (0.48, 0.92)	2E-10	0.51 (0.30, 0.71)	2E-06
**SLEEP LENGTH AND BP: STRATIFIED BY AGE**						
	**AGE**	**Sleep length**	**β (95% CI)**	**p-value**	**β (95% CI)**	**p-value**
**Systolic BP (mmHg)**	**≤50**	≤5	0.86 (0.27, 1.45)	0.0041	0.06 (-0.51, 0.63)	0.834
		6	0.20 (-0.10, 0.5)	0.196	-0.23 (-0.53, 0.07)	0.126
		7	Reference group (β-estimate = 0)			
		8	0.09 (-0.18, 0.35)	0.517	0.23 (-0.02, 0.49)	0.0757

		≥9	0.49 (-0.05, 1.02)	0.0755	0.36 (-0.16, 0.88)	0.176
	>50	≤5	0.86 (0.47, 1.26)	2E-05	0.39 (0, 0.78)	0.0511
		6	0.10 (-0.13, 0.33)	0.394	-0.19 (-0.41, 0.04)	0.109
		7	Reference group (β-estimate = 0)			
		8	0.35 (0.15, 0.55)	0.0006	0.36 (0.16, 0.55)	0.0004
		≥9	0.93 (0.60, 1.26)	3E-08	0.69 (0.36, 1.01)	4E-05
**Diastolic BP (mmHg)**	**≤50**	≤5	0.99 (0.60, 1.37)	5E-07	0.29 (-0.08, 0.65)	0.127
		6	0.37 (0.18, 0.57)	0.0002	-0.01 (-0.20, 0.18)	0.943
		7	Reference group (β-estimate = 0)			
		8	0.13 (-0.04, 0.3)	0.126	0.26 (0.09, 0.42)	0.0021
		≥9	0.77 (0.42, 1.12)	2E-05	0.67 (0.34, 1.00)	8E-05
	**>50**	≤5	0.73 (0.51, 0.94)	5E-11	0.31 (0.10, 0.52)	0.0039
		6	0.23 (0.11, 0.36)	0.0003	-0.01 (-0.13, 0.11)	0.856
		7	Reference group (β-estimate = 0)			
		8	0.30 (0.19, 0.40)	9E-08	0.30 (0.19, 0.40)	3E-08
		≥9	0.77 (0.59, 0.95)	5E-17	0.54 (0.37, 0.72)	1E-09

Supplementary Table S5. β-estimate ± 95% CI for the association between sleep quality and BP when stratified by BMI.

SLEEP QUALITY AND BP: STRATIFIED BY BMI								
			**Baseline model**		**AH free model**		**CRP adj model**	
	**BMI CATEGORY**	**Sleep quality score**	**β (95% CI)**	**p-value**	**β (95% CI)**	**p-value**	**β (95% CI)**	**p-value**
**Systolic BP (mmHg)**	**≤25**	4 to 5	Reference group (β-estimate = 0)					
		2 to 3	0.04 (-0.23, 0.30)	0.796	-0.04 (-0.30, 0.22)	0.758	-0.02 (-0.29, 0.26)	0.916
		0 to 1	-0.07 (-0.53, 0.40)	0.786	-0.24 (-0.70, 0.22)	0.307	-0.27 (-0.75, 0.21)	0.274
	**25-<30**	4 to 5	Reference group (β-estimate = 0)					
		2 to 3	0.12 (-0.14, 0.37)	0.381	-0.12 (-0.38, 0.14)	0.354	0.06 (-0.20, 0.32)	0.645
		0 to 1	0.22 (-0.15, 0.59)	0.247	-0.21 (-0.59, 0.17)	0.277	0.07 (-0.31, 0.44)	0.734
	**≥30**	4 to 5	Reference group (β-estimate = 0)					
		2 to 3	0.56 (0.17, 0.96)	0.0056	0.29 (-0.14, 0.71)	0.187	0.45 (0.04, 0.86)	0.0297
		0 to 1	0.45 (-0.05, 0.95)	0.0758	-0.07 (-0.61, 0.48)	0.815	0.19 (-0.32, 0.70)	0.467
**Diastolic BP (mmHg)**	**≤25**	4 to 5	Reference group (β-estimate = 0)					
		2 to 3	0.20 (0.05, 0.35)	0.008	0.16 (0.02, 0.31)	0.0263	0.17 (0.02, 0.32)	0.0262
		0 to 1	0.40 (0.13, 0.66)	0.003	0.26 (0, 0.52)	0.0466	0.29 (0.02, 0.56)	0.0322
	**25-<30**	4 to 5	Reference group (β-estimate = 0)					
		2 to 3	0.39 (0.24, 0.53)	2E-07	0.22 (0.08, 0.37)	0.0027	0.36 (0.21, 0.51)	2E-06
		0 to 1	0.48 (0.27, 0.68)	8E-06	0.24 (0.03, 0.45)	0.0274	0.39 (0.18, 0.60)	0.0003
	**≥30**	4 to 5	Reference group (β-estimate = 0)					
		2 to 3	0.62 (0.39, 0.84)	9E-08	0.37 (0.13, 0.61)	0.0028	0.55 (0.32, 0.78)	3E-06
		0 to 1	0.81 (0.53, 1.10)	2E-08	0.43 (0.12, 0.74)	0.0066	0.66 (0.37, 0.94)	9E-06

Supplementary Table S6A. β-estimate ± 95% CI for the association between sleep quality and BP when stratified by sex and age.

RELATIONSHIP BETWEEN SLEEP QUALITY AND BLOOD PRESSURE: STRATIFIED BY SEX										
			**Baseline model**		**BMI adj model**		**AH free model**		**AH free BMI adj model**	
	**SEX**	**Sleep quality score**	**β (95% CI)**	**p-value**	**β (95% CI)**	**p-value**	**β (95% CI)**	**p-value**	**β (95% CI)**	**p-value**
**Systolic BP (mmHg)**	**Males**	4 to 5	Reference group (β-estimate = 0)							
		2 to 3	0.44 (0.20, 0.69)	0.000382	-0.18 (-0.42, 0.06)	0.138	0.094 (-0.15, 0.34)	0.455	-0.40 (-0.64, -0.16)	0.0012
		0 to 1	0.81 (0.47, 1.14)	0.00000237	-0.61 (-0.94, -0.28)	0.000314	0.186 (-0.16, 0.54)	0.297	-0.94 (-1.29, -0.06)	9E-08
	**Females**	4 to 5	Reference group (β-estimate = 0)							
		2 to 3	1.01 (0.78, 1.25)	6.41E-17	0.29 (0.06, 0.52)	0.0135	0.704 (0.47, 0.94)	3E-09	0.12 (-0.11, 0.34)	0.325
		0 to 1	2.47 (2.10, 2.84)	6.42E-39	0.38 (0.02, 0.75)	0.0392	1.736 (1.36, 2.11)	1E-19	-0.02 (-0.39, 0.35)	0.92
**Diastolic BP (mmHg)**	**Males**	4 to 5	Reference group (β-estimate = 0)							
		2 to 3	0.76 (0.61, 0.90)	1.63E-24	0.26 (0.12, 0.40)	0.000288	0.52 (0.37, 0.67)	6E-12	0.09 (-0.05, 0.24)	0.199
		0 to 1	1.27 (1.07, 1.47)	1.31E-35	0.14 (-0.05, 0.34)	0.149	0.862 (0.65, 1.07)	9E-16	-0.11 (-0.32, 0.09)	0.273
	**Females**	4 to 5	Reference group (β-estimate = 0)							
		2 to 3	0.99 (0.85, 1.12)	3.45E-47	0.37 (0.25, 0.50)	7.49E-09	0.773 (0.64, 0.90)	6E-31	0.25 (0.13, 0.38)	7E-05
		0 to 1	2.30 (2.09, 2.51)	8.05E-103	0.51 (0.31, 0.71)	0.000000485	1.855 (1.64, 2.07)	9E-67	0.29 (0.09, 0.49)	0.0044
**RELATIONSHIP BETWEEN SLEEP QUALITY AND BLOOD PRESSURE: STRATIFIED BY AGE**										
	**AGE**	**Sleep quality score**	**β (95% CI)**	**p-value**	**β (95% CI)**	**p-value**	**β (95% CI)**	**p-value**	**β (95% CI)**	**p-value**
**Systolic BP (mmHg)**	**>50**	4 to 5	Reference group (β-estimate = 0)							
		2 to 3	1.04 (0.78, 1.30)	1.1E-14	0.23 (-0.02, 0.48)	0.076	0.801 (0.55, 1.05)	6E-10	0.12 (-0.13, 0.36)	0.352

		0 to 1	2.36 (1.92, 2.80)	9.98E-26	-0.01 (-0.43, 0.42)	0.977	1.689 (1.26, 2.12)	2E-14	-0.31 (-0.73, 0.11)	0.145
	**≤50**	4 to 5	Reference group (β-estimate = 0)							
		2 to 3	0.73 (0.52, 0.95)	4.38E-11	0.11 (-0.10, 0.32)	0.316	0.353 (0.13, 0.58)	0.001 9	-0.13 (-0.35, 0.09)	0.248
		0 to 1	1.56 (1.26, 1.87)	1.11E-23	0.02 (-0.28, 0.32)	0.911	0.857 (0.54, 1.18)	2E-07	-0.38 (-0.69, -0.06)	0.021 2
**Diastolic BP (mmHg)**	**>50**	4 to 5	Reference group (β-estimate = 0)							
		2 to 3	1.14 (0.97, 1.32)	6.87E-38	0.44 (0.27, 0.60)	0.00000015	0.975 (0.81, 1.14)	5E-30	0.35 (0.20, 0.51)	1E-05
		0 to 1	2.50 (2.21, 2.79)	2.69E-63	0.45 (0.17, 0.72)	0.00137	2.032 (1.75, 2.32)	2E-44	0.23 (-0.04, 0.50)	0.088 9
	**≤50**	4 to 5	Reference group (β-estimate = 0)							
		2 to 3	0.73 (0.61, 0.85)	2.18E-33	0.23 (0.12, 0.35)	0.000064	0.468 (0.35, 0.59)	3E-14	0.06 (-0.06, 0.17)	0.33
		0 to 1	1.50 (1.33, 1.66)	1.02E-68	0.24 (0.08, 0.40)	0.0032	1.052 (0.88, 1.23)	4E-32	-0.01 (-0.18, 0.16)	0.877

Supplementary Table S6B. β-estimate ± 95% CI for the association between sleep quality and BP when stratified by sex and age.

RELATIONSHIP BETWEEN SLEEP QUALITY AND BLOOD PRESSURE: STRATIFIED BY SEX						
			CRP adj model		BMI and CRP adj model	
	**SEX**	**Sleep quality score**	**β (95% CI)**	**p-value**	**β (95% CI)**	**p-value**
**Systolic BP (mmHg)**	**Males**	4 to 5	Reference group (β-estimate = 0)			
		2 to 3	0.27 (0.02, 0.52)	0.0316	-0.18 (-0.43, 0.06)	0.141
		0 to 1	0.42 (0.07, 0.76)	0.0176	-0.66 (-1.00, -0.32)	0.000134
	**Females**	4 to 5	Reference group (β-estimate = 0)			
		2 to 3	0.63 (0.39, 0.87)	0.000000248	0.21 (-0.03, 0.450)	0.0858
		0 to 1	1.45 (1.07, 1.83)	4.64E-14	0.15 (-0.22, 0.53)	0.421
**Diastolic BP (mmHg)**	**Males**	4 to 5	Reference group (β-estimate = 0)			
		2 to 3	0.62 (0.47, 0.77)	1.67E-16	0.25 (0.11, 0.39)	0.000614
		0 to 1	0.98 (0.78, 1.18)	3.73E-21	0.11 (-0.09, 0.31)	0.274
	**Females**	4 to 5	Reference group (β-estimate = 0)			
		2 to 3	0.72 (0.58, 0.85)	1.6E-25	0.33 (0.20, 0.46)	0.000000467
		0 to 1	1.58 (1.37, 1.79	8.17E-49	0.39 (0.19, 0.60)	0.000158
**RELATIONSHIP BETWEEN SLEEP QUALITY AND BLOOD PRESSURE: STRATIFIED BY AGE**						
	**AGE**	**Sleep quality score**	**β (95% CI)**	**p-value**	**β (95% CI)**	**p-value**
**Systolic BP (mmHg)**	**>50**	4 to 5	Reference group (β-estimate = 0)			
		2 to 3	0.71 (0.44, 0.97)	0.000000215	0.20 (-0.06, 0.46)	0.128
		0 to 1	1.47 (1.02, 1.97)	1.22E-10	-0.11 (-0.55, 0.33)	0.621
	**≤50**	4 to 5	Reference group (β-estimate = 0)			
		2 to 3	0.45 (0.23, 0.67)	0.0000704	0.05 (-0.17, 0.26)	0.684
		0 to 1	0.89 (0.58, 1.20)	2.31E-08	-0.15 (-0.46, 0.16)	0.341
**Diastolic BP (mmHg)**	**>50**	4 to 5	Reference group (β-estimate = 0)			
		2 to 3	0.82 (0.65, 0.99)	2.67E-20	0.38 (0.21, 0.54)	0.00000805
		0 to 1	1.69 (1.40, 1.98)	8.95E-30	0.32 (0.04, 0.60)	0.0253
	**≤50**	4 to 5	Reference group (β-estimate = 0)			
		2 to 3	0.57 (0.45, 0.69)	3.19E-20	0.22 (0.11, 0.34)	0.000199
		0 to 1	1.09 (0.92, 1.26)	1.96E-36	0.19 (0.02, 0.35)	0.0255

Supplementary Table S7. β-estimate ± 95% CI for the association between shift work and BP when stratified by BMI.

RELATIONSHIP BETWEEN SHIFT WORK AND BP: STRATIFIED BY BMI								
	BMI CATEGORY		Baseline model		AH free model		CRP adj model	
		Shift work	β (95% CI)	p-value	β (95% CI)	p-value	β (95% CI)	p-value
Systolic BP (mmHg)	≤25	No shift work	Reference group (β-estimate = 0)					
		Day-shift	-0.58 (-1.09, -0.06)	0.0284	-0.54 (-1.04, -0.03)	0.0366	-0.53 (-1.06, 0.00)	0.0485
		Mixed-shift	-0.10 (-0.81, 0.62)	0.794	-0.06 (-0.76, 0.64)	0.872	-0.14 (-0.87, 0.60)	0.716
		Night-shift	-1.16 (-2.47, 0.15)	0.0819	-0.77 (-2.03, 0.49)	0.228	-1.35 (-2.67, -0.02)	0.0473
		Permanent night-shift	0.64 (-0.50, 1.77)	0.274	0.58 (-0.52, 1.69)	0.301	0.55 (-0.61, 1.72)	0.352
	25-<30	No shift work	Reference group (β-estimate = 0)					
		Day-shift	-0.62 (-1.08, -0.17)	0.0072	-0.53 (-0.98, -0.09)	0.0197	-0.68 (-1.14, -0.21)	0.0044
		Mixed-shift	-0.28 (-0.85, 0.30)	0.349	-0.19 (-0.76, 0.37)	0.504	-0.32 (-0.91, 0.27)	0.287
		Night-shift	-0.70 (-1.72, 0.32)	0.18	-0.31 (-1.31, 0.70)	0.55	-0.72 (-1.76, 0.33)	0.178
		Permanent night-shift	-0.20 (-1.03, 0.63)	0.634	-0.03 (-0.85, 0.79)	0.949	-0.33 (-1.18, 0.52)	0.448
	≥30	No shift work	Reference group (β-estimate = 0)					
		Day-shift	0.01 (-0.61, 0.62)	0.988	-0.19 (-0.83, 0.45)	0.553	-0.05 (-0.67, 0.58)	0.882
		Mixed-shift	-0.24 (-0.99, 0.51)	0.528	-0.20 (-0.98, 0.58)	0.616	-0.28 (-1.05, 0.48)	0.467
		Night-shift	-0.70 (-2.00, 0.60)	0.293	-0.70 (-2.06, 0.66)	0.311	-0.79 (-2.11, 0.53)	0.243
		Permanent night-shift	0.54 (-0.52, 1.61)	0.319	0.62 (-0.48, 1.73)	0.27	0.06 (-1.04, 1.15)	0.922
Diastolic BP (mmHg)	≤25	No shift work	Reference group (β-estimate = 0)					
		Day-shift	-0.21 (-0.51, 0.09)	0.174	-0.26 (-0.55, 0.04)	0.0874	-0.20 (-0.51, 0.11)	0.199
		Mixed-shift	-0.04 (-0.46, 0.39)	0.863	-0.07 (-0.48, 0.34)	0.745	-0.10 (-0.53, 0.33)	0.647
		Night-shift	-0.47 (-1.23, 0.30)	0.233	-0.25 (-0.99, 0.49)	0.51	-0.55 (-1.33, 0.23)	0.167
		Permanent night-shift	0.28 (-0.39, 0.95)	0.413	0.31 (-0.34, 0.96)	0.354	0.25 (-0.44, 0.93)	0.482
	25-<30	No shift work	Reference group (β-estimate = 0)					
		Day-shift	-0.23 (-0.50, 0.04)	0.0905	-0.14 (-0.41, 0.13)	0.3	-0.30 (-0.57, -0.02)	0.0355
		Mixed-shift	0.10 (-0.24, 0.44)	0.554	0.13 (-0.21, 0.46)	0.464	0.05 (-0.30, 0.40)	0.785
		Night-shift	-0.10 (-0.71, 0.51)	0.753	0.06 (-0.54, 0.66)	0.842	-0.18 (-0.80, 0.44)	0.562
		Permanent night-shift	0.003 (-0.49, 0.49)	0.989	0.03 (-0.45, 0.52)	0.891	-0.09 (-0.59, 0.41)	0.722
	≥30	No shift work	Reference group (β-estimate = 0)					

		Day-shift	0.09 (-0.27, 0.45)	0.633	-0.02 (-0.40, 0.35)	0.905	0.03 (-0.33, 0.40)	0.871
		Mixed-shift	0.01 (-0.43, 0.45)	0.953	-0.08 (-0.53, 0.38)	0.747	0.04 (-0.40, 0.49)	0.847
		Night-shift	-0.31 (-1.07, 0.45)	0.429	-0.45 (-1.24, 0.35)	0.272	-0.33 (-1.11, 0.44)	0.397
		Permanent night-shift	0.68 (0.06, 1.31)	0.0329	0.77 (0.12, 1.42)	0.0196	0.44 (-0.21, 1.08)	0.183

Supplementary Table S8. β-estimate ± 95% CI for the association between shift work and BP when stratified by BMI and sex.

RELATIONSHIP BETWEEN SHIFT WORK AND BP: STRATIFIED BY BMI AND SEX				
		Baseline model		
	**BMI AND SEX CATEGORY**	**Shift work**	**β (95% CI)**	**p-value**
**Systolic BP (mmHg)**	**Males ≤25**	No shift work	Reference group (β-estimate = 0)	
		Day-shift	-0.75 (-1.40, -0.09)	0.0256
		Mixed-shift	-0.82 (-1.82, 0.17)	0.106
		Night-shift	-0.97 (-2.86, 0.93)	0.317
		Permanent night-shift	-0.49 (-2.16, 1.18)	0.567
	**Males >25**	No shift work	Reference group (β-estimate = 0)	
		Day-shift	-0.24 (-0.82, 0.34)	0.416
		Mixed-shift	-0.64 (-1.54, 0.25)	0.159
		Night-shift	-0.78 (-2.40, 0.84)	0.347
		Permanent night-shift	0.23 (-1.09, 1.55)	0.734
	**Females ≤25**	No shift work	Reference group (β-estimate = 0)	
		Day-shift	-0.32 (-1.15, 0.51)	0.445
		Mixed-shift	0.52 (-0.51, 1.55)	0.321
		Night-shift	-1.55 (-3.34, 0.23)	0.0881
		Permanent night-shift	1.37 (-0.17, 2.90)	0.0803
	**Females >25**	No shift work	Reference group (β-estimate = 0)	
		Day-shift	-0.18 (-0.66, 0.29)	0.449
		Mixed-shift	0.25 (-0.28, 0.78)	0.349
		Night-shift	-0.33 (-1.26, 0.59)	0.478
		Permanent night-shift	0.31 (-0.45, 1.06)	0.424
**Diastolic BP (mmHg)**	**Males ≤25**	No shift work	Reference group (β-estimate = 0)	
		Day-shift	-0.26 (-0.64, 0.12)	0.179
		Mixed-shift	-0.41 (-0.98, 0.17)	0.163
		Night-shift	-0.49 (-1.57, 0.60)	0.377
		Permanent night-shift	-0.76 (-1.72, 0.20)	0.121
	**Males >25**	No shift work	Reference group (β-estimate = 0)	
		Day-shift	-0.13 (-0.47, 0.20)	0.441
		Mixed-shift	-0.12 (-0.63, 0.40)	0.663
		Night-shift	-0.27 (-1.21, 0.67)	0.572
		Permanent night-shift	0.07 (-0.69, 0.83)	0.859
	**Females ≤25**	No shift work	Reference group (β-estimate = 0)	
		Day-shift	-0.16 (-0.67, 0.35)	0.538
		Mixed-shift	0.31 (-0.32, 0.94)	0.337
		Night-shift	-0.53 (-1.62, 0.56)	0.337
		Permanent night-shift	1.15 (0.20, 2.09)	0.0171
	**Females >25**	No shift work	Reference group (β-estimate = 0)	
		Day-shift	0.19 (-0.10, 0.48)	0.202
		Mixed-shift	0.53 (0.21, 0.86)	0.0014
		Night-shift	0.25 (-0.32, 0.82)	0.384
		Permanent night-shift	0.70 (0.23, 1.16)	0.0032

Supplementary Table S9A. β-estimate ± 95% CI for the association between shift work and BP when stratified by sex and age.

RELATIONSHIP BETWEEN SHIFT WORK AND BP: STRATIFIED BY SEX								
			**Baseline model**		**BMI adj model**		**AH free model**	
	**SEX**	**Shift work**	**β (95% CI)**	**p-value**	**β (95% CI)**	**p-value**	**β (95% CI)**	**p-value**
**Systolic BP (mmHg)**	**Males**	No shift work	Reference group (β-estimate = 0)					
		Day-shift	-0.03 (-0.45, 0.39)	0.89	-0.41 (-0.82, 0.00)	0.0477	-0.089 (-0.51, 0.33)	6.79E-01
		Mixed-shift	0.75 (0.27, 1.22)	0.0022	-0.08 (-0.55, 0.38)	0.724	0.631 (0.15, 1.11)	9.49E-03
		Night-shift	-0.20 (-1.03, 0.63)	0.641	-0.90 (-1.71, -0.09)	0.0294	-0.036 (-0.86, 0.79)	9.32E-01
		Permanent night-shift	0.99 (0.30, 1.67)	0.00482	0.27 (-0.40, 0.94)	0.426	0.982 (0.30, 1.67)	4.93E-03
	**Females**	No shift work	Reference group (β-estimate = 0)					
		Day-shift	-0.12 (-0.57, 0.32)	0.585	-0.60 (-1.03, -0.17)	0.006	-0.22 (-0.66, 0.21)	3.11E-01
		Mixed-shift	-0.36 (-1.04, 0.32)	0.294	-1.01 (-1.66, -0.35)	0.0027	-0.25 (-0.90, 0.41)	4.65E-01
		Night-shift	-0.36 (-1.61, 0.89)	0.573	-1.19 (-2.40, 0.03)	0.0552	-0.34 (-1.56, 0.88)	5.84E-01
		Permanent night-shift	0.63 (-0.43, 1.68)	0.243	-0.60 (-1.62, 0.42)	0.246	0.59 (-0.43, 1.62)	2.58E-01
**Diastolic BP (mmHg)**	**Males**	No shift work	Reference group (β-estimate = 0)					
		Day-shift	0.27 (0.01, 0.53)	0.0448	-0.06 (-0.31, 0.19)	0.63	0.23 (-0.04, 0.49)	0.091
		Mixed-shift	0.88 (0.59, 1.18)	6E-09	0.17 (-0.11, 0.45)	0.236	0.76 (0.46, 1.05)	7.2E-07
		Night-shift	0.42 (-0.10, 0.94)	0.11	-0.18 (-0.67, 0.31)	0.472	0.38 (-0.14, 0.89)	0.155
		Permanent night-shift	1.20 (0.77, 1.63)	4E-08	0.60 (0.19, 1.00)	0.0039	1.23 (0.80, 1.65)	1.96E-08
	**Females**	No shift work	Reference group (β-estimate = 0)					
		Day-shift	0.09 (-0.18, 0.35)	0.517	-0.32 (-0.56, -0.08)	0.0099	0.01 (-0.25, 0.27)	0.939
		Mixed-shift	0.06 (-0.34, 0.46)	0.763	-0.48 (-0.85, -0.11)	0.0118	0.06 (-0.33, 0.45)	0.76
		Night-shift	0.01 (-0.73, 0.75)	0.978	-0.65 (-1.34, 0.04)	0.065	0.06 (-0.66, 0.78)	0.875
		Permanent night-shift	0.29 (-0.33, 0.91)	0.352	-0.71 (-1.29, -0.13)	0.0159	0.22 (-0.38, 0.83)	0.47
**RELATIONSHIP BETWEEN SHIFT WORK AND BP: STRATIFIED BY AGE**								
	**AGE**	**Shift work**	**β (95% CI)**	**p-value**	**β (95% CI)**	**p-value**	**β (95% CI)**	**p-value**
**Systolic BP (mmHg)**	**≤50**	No shift work	Reference group (β-estimate = 0)					
		Day-shift	-0.19 (-0.62, 0.23)	0.368	-0.67 (-1.07, -0.26)	0.0012	-0.24 (-0.65, 0.17)	0.246
		Mixed-shift	0.04 (-0.47, 0.54)	0.886	-0.74 (-1.22, -0.26)	0.0027	0.01 (-0.47, 0.50)	0.954
		Night-shift	-0.65 (-1.54, 0.23)	0.147	-1.28 (-2.13, -0.43)	0.0031	-0.58 (-1.44, 0.28)	0.183
		Permanent night-shift	0.62 (-0.13, 1.37)	0.107	-0.16 (-0.88, 0.56)	0.663	0.63 (-0.10, 1.36)	0.0929
	**>50**	No shift work	Reference group (β-estimate = 0)					

		Day-shift	-0.15 (-0.58, 0.29)	0.502	-0.54 (-0.96, -0.11)	0.013	-0.19 (-0.63, 0.25)	0.399
		Mixed-shift	-0.07 (-0.67, 0.54)	0.833	-0.79 (-1.38, -0.21)	0.0081	0.02 (-0.59, 0.64)	0.947
		Night-shift	-0.65 (-1.73, 0.43)	0.242	-1.47 (-2.52, -0.42)	0.0063	-0.17 (-1.28, 0.93)	0.759
		Permanent night-shift	0.43 (-0.45, 1.30)	0.34	-0.51 (-1.37, 0.34)	0.241	0.59 (-0.30, 1.49)	0.193
**Diastolic BP (mmHg)**	**≤50**	No shift work	Reference group (β-estimate = 0)					
		Day-shift	0.11 (-0.18, 0.38)	0.464	-0.33 (-0.59, -0.07)	0.0138	0.12 (-0.15, 0.39)	0.391
		Mixed-shift	0.44 (0.11, 0.77)	0.0093	-0.24 (-0.55, 0.07)	0.128	0.40 (0.08, 0.73)	0.0148
		Night-shift	0.27 (-0.31, 0.86)	0.361	-0.26 (-0.81, 0.28)	0.34	0.26 (-0.31, 0.83)	0.372
		Permanent night-shift	0.85 (0.35, 1.35)	0.0008	0.16 (-0.3, 0.62)	0.49	0.83 (0.35, 1.32)	0.000722
	**>50**	No shift work	Reference group (β-estimate = 0)					
		Day-shift	0.20 (-0.05, 0.44)	0.117	-0.11 (-0.35, 0.12)	0.347	0.10 (-0.15, 0.35)	0.425
		Mixed-shift	0.64 (0.30, 0.98)	0.0003	0.04 (-0.28, 0.36)	0.819	0.56 (0.22, 0.91)	0.00144
		Night-shift	0.17 (-0.44, 0.78)	0.58	-0.49 (-1.07, 0.09)	0.0979	0.25 (-0.37, 0.87)	0.434
		Permanent night-shift	0.82 (0.33, 1.32)	0.0011	0.10 (-0.37, 0.56)	0.692	0.85 (0.35, 1.35)	0.000847

Supplementary Table S9B. β-estimate ± 95% CI for the association between shift work and BP when stratified by sex and age.

RELATIONSHIP BETWEEN SHIFT WORK AND BP: STRATIFIED BY SEX								
			**AH free BMI adj model**		**CRP adj model**		**BMI and CRP adj model**	
	**SEX**	**Shift work**	**β (95% CI)**	**p-value**	**β (95% CI)**	**p-value**	**β (95% CI)**	**p-value**
**Systolic BP (mmHg)**	**Males**	No shift work	Reference group (β-estimate = 0)					
		Day-shift	-0.36 (-0.78, 0.05)	0.0828	-0.10 (-0.52, 0.33)	0.663	-0.40 (-0.81, 0.02)	0.0633
		Mixed-shift	-0.06 (-0.53, 0.41)	0.8	0.48 (-0.01, 0.97)	0.0529	-0.12 (-0.60, 0.36)	0.618
		Night-shift	-0.56 (-1.37, 0.25)	0.174	-0.43 (-1.27, 0.41)	0.317	-0.91 (-1.74, -0.09)	0.03
		Permanent night-shift	0.36 (-0.31, 1.03)	0.294	0.53 (-0.16, 1.23)	0.134	0.07 (-0.62, 0.75)	0.848
	**Females**	No shift work	Reference group (β-estimate = 0)					
		Day-shift	-0.64 (-1.06, -0.21)	0.0031	-0.40 (-0.85, 0.04)	0.0764	-0.64 (-1.08, -0.20)	0.0044
		Mixed-shift	-0.81 (-1.45, -0.17)	0.0135	-0.57 (-1.25, 0.12)	0.104	-0.98 (-1.65, -0.31)	0.0044
		Night-shift	-1.01 (-2.19, 0.18)	0.0966	-0.89 (-2.14, 0.37)	0.168	-1.28 (-2.52, -0.05)	0.0421
		Permanent night-shift	-0.40 (-1.40, 0.60)	0.433	0.02 (-1.04, 1.08)	0.967	-0.75 (-1.79, 0.29)	0.158
**Diastolic BP (mmHg)**	**Males**	No shift work	Reference group (β-estimate = 0)					
		Day-shift	-0.03 (-0.28, 0.23)	0.847	0.18 (-0.09, 0.44)	0.189	-0.08 (-0.34, 0.17)	0.516
		Mixed-shift	0.13 (-0.16, 0.41)	0.392	0.66 (0.36, 0.96)	2E-05	0.13 (-0.16, 0.42)	0.365
		Night-shift	-0.11 (-0.60, 0.39)	0.669	0.18 (-0.34, 0.70)	0.496	-0.24 (-0.75, 0.26)	0.342
		Permanent night-shift	0.66 (0.25, 1.07)	0.0015	0.89 (0.46, 1.32)	5E-05	0.49 (0.08, 0.91)	0.02
	**Females**	No shift work	Reference group (β-estimate = 0)					
		Day-shift	-0.35 (-0.59, -0.11)	0.0038	-0.14 (-0.40, 0.12)	0.281	-0.36 (-0.60, -0.11)	0.0052
		Mixed-shift	-0.43 (-0.79, -0.06)	0.0215	-0.08 (-0.48, 0.32)	0.701	-0.44 (-0.82, -0.06)	0.0234
		Night-shift	-0.50 (-1.18, 0.17)	0.143	-0.34 (-1.07, 0.40)	0.369	-0.65 (-1.35, 0.05)	0.0691
		Permanent night-shift	-0.62 (-1.19, -0.05)	0.0318	-0.16 (-0.78, 0.46)	0.609	-0.81 (-1.40, -0.22)	0.0069
**RELATIONSHIP BETWEEN SHIFT WORK AND BP: STRATIFIED BY AGE**								
	**AGE**	**Shift work**	**β (95% CI)**	**p-value**	**β (95% CI)**	**p-value**	**β (95% CI)**	**p-value**
**Systolic BP (mmHg)**	**≤50**	No shift work	Reference group (β-estimate = 0)					
		Day-shift	-0.65 (-1.05, -0.26)	0.0013	-0.35 (-0.77, 0.08)	0.112	-0.67 (-1.08, -0.25)	0.0016
		Mixed-shift	-0.66 (-1.13, -0.18)	0.0063	-0.14 (-0.65, 0.36)	0.581	-0.71 (-1.21, -0.22)	0.0046
		Night-shift	-1.13 (-1.95, -0.30)	0.0076	-0.85 (-1.74, 0.04)	0.062	-1.25 (-2.11, -0.38)	0.0047
		Permanent night-shift	-0.08 (-0.78, 0.63)	0.826	0.10 (-0.66, 0.85)	0.8	-0.38 (-1.11, 0.36)	0.319
	**>50**	No shift work	Reference group (β-estimate = 0)					

		Day-shift	-0.48 (-0.91, -0.04)	0.0309	-0.33 (-0.77, 0.11)	0.14	-0.57 (-1.00, -0.13)	0.0107
		Mixed-shift	-0.59 (-1.19, 0.01)	0.0554	-0.38 (-0.99, 0.23)	0.217	-0.82 (-1.42, -0.22)	0.0073
		Night-shift	-0.77 (-1.85, 0.32)	0.167	-1.15 (-2.24, -0.06)	0.0381	-1.60 (-2.67, -0.52)	0.0035
		Permanent night-shift	-0.17 (-1.05, 0.70)	0.698	-0.04 (-0.92, 0.85)	0.937	-0.63 (-1.51, 0.24)	0.156
**Diastolic BP (mmHg)**	**≤50**	No shift work	Reference group (β-estimate = 0)					
		Day-shift	-0.27 (-0.52, -0.01)	0.0397	-0.04 (-0.32, 0.23)	0.756	-0.34 (-0.60, -0.07)	0.0125
		Mixed-shift	-0.20 (-0.51, 0.10)	0.185	0.25 (-0.08, 0.58)	0.139	-0.25 (-0.56, 0.07)	0.128
		Night-shift	-0.24 (-0.77, 0.29)	0.38	0.04 (-0.54, 0.62)	0.883	-0.29 (-0.84, 0.26)	0.301
		Permanent night-shift	0.18 (-0.27, 0.63)	0.436	0.52 (0.03, 1.02)	0.0382	0.11 (-0.37, 0.57)	0.662
	**>50**	No shift work	Reference group (β-estimate = 0)					
		Day-shift	-0.14 (-0.38, 0.10)	0.252	0.05 (-0.20, 0.30)	0.68	-0.15 (-0.39, 0.09)	0.223
		Mixed-shift	0.03 (-0.30, 0.36)	0.851	0.44 (0.10, 0.79)	0.0113	0.05 (-0.28, 0.38)	0.777
		Night-shift	-0.25 (-0.85, 0.34)	0.403	-0.13 (-0.74, 0.48)	0.68	-0.52 (-1.11, 0.07)	0.0834
		Permanent night-shift	0.22 (-0.25, 0.70)	0.36	0.46 (-0.04, 0.95)	0.0735	-0.03 (-0.51, 0.45)	0.896

Supplementary Table S10A. β-estimate ± 95% CI for the association between sleep length x shift work and BP.

INTERACTION BETWEEN SLEEP LENGTH x SHIFT WORK AND BP							
		Baseline model		BMI adj model		AH free model	
	**INTERACTION**	**β (95% CI)**	**p-value**	**β (95% CI)**	**p-value**	**β (95% CI)**	**p-value**
**Systolic BP (mmHg)**	≤5 hours/day x Day shift work	1.50 (0.26, 2.74)	0.0181	0.25 (-0.95, 1.46)	0.682	1.23 (-0.03, 2.49)	0.056281
	≤5 hours/day x Mixed shift work	1.47 (-0.05, 2.98)	0.0583	-0.27 (-1.74, 1.21)	0.724	1.33 (-0.18, 2.85)	0.084883
	≤5 hours/day x Night shift work	2.00 (-0.57, 4.57)	0.127	0.25 (-2.24, 2.75)	0.844	1.88 (-0.77, 4.52)	0.164679
	≤5 hours/day x No shift work	0.99 (0.52, 1.45)	4E-05	0.02 (-0.44, 0.47)	0.95	0.83 (0.36, 1.30)	0.000505
	≤5 hours/day x Permanent night-shift	2.57 (0.80, 4.34)	0.0045	1.15 (-0.57, 2.87)	0.188	1.33 (-0.48, 3.13)	0.149554
	≥9 hours/day x Day shift work	0.57 (-0.76, 1.91)	0.4	-0.26 (-1.56, 1.04)	0.692	-0.02 (-1.34, 1.31)	0.982376
	≥9 hours/day x Mixed shift work	-0.94 (-2.77, 0.89)	0.313	-1.58 (-3.35, 0.19)	0.0803	-1.21 (-3.03, 0.61)	0.192931
	≥9 hours/day x Night shift work	-0.66 (-3.71, 2.39)	0.673	-1.02 (-3.97, 1.94)	0.5	-1.02 (-4.08, 2.05)	0.516177
	≥9 hours/day x No shift work	1.12 (0.65, 1.59)	3E-06	0.77 (0.32, 1.23)	0.0009	0.68 (0.21, 1.15)	0.004308
	≥9 hours/day x Permanent night-shift	1.78 (-0.76, 4.31)	0.17	0.51 (-1.94, 2.96)	0.682	1.62 (-0.88, 4.12)	0.204079
	6 hours/day x Day shift work	0.58 (-0.04, 1.20)	0.0664	-0.46 (-1.06, 0.14)	0.136	0.53 (-0.09, 1.14)	0.094997
	6 hours/day x Mixed shift work	0.59 (-0.18, 1.36)	0.134	-0.66 (-1.41, 0.09)	0.0855	0.43 (-0.34, 1.20)	0.273301
	6 hours/day x Night shift work	-0.61 (-1.94, 0.37)	0.372	-1.72 (-3.02, -0.43)	0.009	-0.28 (-1.59, 1.04)	0.682731
	6 hours/day x No shift work	0.16 (-0.07, 0.40)	0.17	-0.34 (-0.57, -0.11)	0.0034	0.01 (-0.22, 0.25)	0.907192
	6 hours/day x Permanent night-shift	1.18 (0.10, 2.26)	0.033	-0.07 (-1.12, 0.98)	0.901	0.91 (-0.18, 1.99)	0.10083
	7 hours/day x No shift work	Reference group (β-estimate = 0)					
	7 hours/day x Day shift work	-0.28 (-0.75, 0.19)	0.245	-0.70 (-1.16, -0.25)	0.0026	-0.31 (-0.78, 0.15)	0.186081
	7 hours/day x Mixed shift work	0.51 (-0.10, 1.11)	0.102	-0.19 (-0.78, 0.40)	0.524	0.597 (0, 1.19)	0.049382
	7 hours/day x Night shift work	0.04 (-1.11, 1.18)	0.949	-0.71 (-1.81, 0.40)	0.211	0.16 (-0.96, 1.29)	0.775904
	7 hours/day x Permanent night-shift	0.47 (-0.50, 1.45)	0.34	-0.36 (-1.30, 0.58)	0.453	0.75 (-0.21, 1.71)	0.125058
	8 hours/day x Day shift work	0.06 (-0.53, 0.65)	0.846	-0.15 (-0.72, 0.42)	0.607	-0.23 (-0.81, 0.36)	0.451862
	8 hours/day x Mixed shift work	0.99 (0.18, 1.80)	0.0172	0.24 (-0.55, 1.03)	0.553	0.59 (-0.22, 1.39)	0.153071
	8 hours/day x Night shift work	0.27 (-1.17, 1.72)	0.714	-0.45 (-1.85, 0.96)	0.533	0.14 (-1.28, 1.56)	0.848333

	8 hours/day x No shift work	0.37 (0.16, 0.59)	0.0005	0.41 (0.21, 0.62)	8E-05	0.30 (0.09, 0.51)	0.005145
	8 hours/day x Permanent night-shift	1.52 (0.26, 2.77)	0.0175	0.70 (-0.51, 1.91)	0.257	1.69 (0.46, 2.93)	0.007197
	**INTERACTION**	**β (95% CI)**	**p-value**	**β (95% CI)**	**p-value**	**β (95% CI)**	**p-value**
**Diastolic BP (mmHg)**	≤5 hours/day x Day shift work	1.34 (0.59, 2.09)	0.0005	0.32 (-0.39, 1.02)	0.377	0.99 (0.23, 1.75)	0.0108
	≤5 hours/day x Mixed shift work	1.75 (0.83, 2.67)	0.0002	0.32 (-0.55, 1.18)	0.471	1.29 (0.37, 2.21)	0.0058
	≤5 hours/day x Night shift work	2.37 (0.81, 3.92)	0.0029	0.86 (-0.61, 2.32)	0.251	2.11 (0.51, 3.71)	0.00981
	≤5 hours/day x No shift work	1.02 (0.73, 1.30)	2E-12	0.20 (-0.07, 0.47)	0.139	0.83 (0.54, 1.11)	1.15E-08
	≤5 hours/day x Permanent night-shift	2.29 (1.22, 3.36)	3E-05	1.14 (0.13, 2.15)	0.0266	1.76 (0.67, 2.84)	0.00152
	≥9 hours/day x Day shift work	0.96 (0.15, 1.77)	0.0207	0.26 (-0.51, 1.02)	0.507	0.76 (-0.05, 1.56)	0.0654
	≥9 hours/day x Mixed shift work	0.29 (-0.81, 1.40)	0.604	-0.27 (-1.31, 0.77)	0.613	-0.13 (-1.23, 0.97)	0.815
	≥9 hours/day x Night shift work	0.56 (-1.30, 2.41)	0.555	0.29 (-1.45, 2.03)	0.746	0.34 (-1.53, 2.20)	0.724
	≥9 hours/day x No shift work	1.09 (0.80, 1.37)	6E-14	0.79 (0.52, 1.06)	7E-09	0.82 (0.54, 1.10)	1.44E-08
	≥9 hours/day x Permanent night-shift	1.35 (-0.18, 2.88)	0.0848	0.29 (-1.14, 1.73)	0.689	0.97 (-0.54, 2.48)	0.207
	6 hours/day x Day shift work	0.82 (0.45, 1.20)	2E-05	-0.05 (-0.41, 0.30)	0.764	0.76 (0.39, 1.14)	0.000065
	6 hours/day x Mixed shift work	0.87 (0.41, 1.34)	0.0003	-0.19 (-0.63, 0.25)	0.403	0.69 (0.23, 1.16)	0.00364
	6 hours/day x Night shift work	0.21 (-0.59, 1.02)	0.605	-0.73 (-1.49, 0.03)	0.0603	0.21 (-0.59, 1.01)	0.609
	6 hours/day x No shift work	0.38 (0.24, 0.52)	1E-07	-0.04 (-0.17, 0.10)	0.581	0.28 (0.14, 0.42)	0.000083
	6 hours/day x Permanent night-shift	1.50 (0.84, 2.15)	8E-06	0.46 (-0.16, 1.07)	0.145	1.21 (0.56, 1.87)	0.00028
	7 hours/day x No shift work	Reference group (β-estimate = 0)					
	7 hours/day x Day shift work	0.04 (-0.25, 0.32)	0.799	-0.32 (-0.59, -0.05)	0.0193	-0.02 (-0.30, 0.26)	0.885
	7 hours/day x Mixed shift work	0.70 (0.33, 1.06)	0.0002	0.10 (-0.24, 0.45)	0.557	0.69 (0.32, 1.05)	0.000197
	7 hours/day x Night shift work	0.32 (-0.37, 1.01)	0.362	-0.27 (-0.92, 0.38)	0.412	0.45 (-0.23, 1.13)	0.194
	7 hours/day x Permanent night-shift	0.85 (0.26, 1.44)	0.0045	0.17 (-0.39, 0.72)	0.554	0.98 (0.40, 1.56)	0.000912
	8 hours/day x Day shift work	0.39 (0.03, 0.75)	0.0335	0.21 (-0.13, 0.54)	0.229	0.19 (-0.16, 0.55)	0.292
	8 hours/day x Mixed shift work	0.96 (0.47, 1.45)	0.0001	0.33 (-0.14, 0.79)	0.168	0.71 (0.22, 1.19)	0.00461
	8 hours/day x Night shift work	0.66 (-0.22, 1.53)	0.142	0.04 (-0.78, 0.86)	0.928	0.38 (-0.48, 1.24)	0.39
	8 hours/day x No shift work	0.30 (0.18, 0.43)	3E-06	0.34 (0.21, 0.46)	5E-08	0.22 (0.09, 0.34)	0.0007
	8 hours/day x Permanent night-shift	0.71 (-0.04, 1.47)	0.0642	0.03 (-0.68, 0.74)	0.932	0.83 (0.08, 1.57)	0.03

Supplementary Table S10B. β-estimate ± 95% CI for the association between sleep length x shift work and BP.

INTERACTION BETWEEN SLEEP LENGTH x SHIFT WORK AND BP					
		AH free BMI adj model		BMI and CRP adj model	
	INTERACTION	β (95% CI)	p-value	β (95% CI)	p-value
Systolic BP (mmHg)	≤5 hours/day x Day shift work	0.24 (-0.99, 1.47)	0.697835	0.45 (-0.78, 1.69)	0.4715
	≤5 hours/day x Mixed shift work	-0.05 (-1.53, 1.43)	0.948349	-0.26 (-1.77, 1.25)	0.7375
	≤5 hours/day x Night shift work	0.53 (-2.06, 3.11)	0.688631	0.48 (-2.08, 3.04)	0.7151
	≤5 hours/day x No shift work	0.03 (-0.43, 0.48)	0.907194	-0.05 (-0.51, 0.42)	0.8406
	≤5 hours/day x Permanent night-shift	0.22 (-1.54, 1.98)	0.80706	0.76 (-1.02, 2.54)	0.4026
	≥9 hours/day x Day shift work	-0.64 (-1.94, 0.65)	0.3315	-0.43 (-1.76, 0.91)	0.5333
	≥9 hours/day x Mixed shift work	-1.63 (-3.41, 0.14)	0.070814	-1.66 (-3.45, 0.13)	0.0694
	≥9 hours/day x Night shift work	-0.90 (-3.88, 2.09)	0.55693	-1.30 (-4.35, 1.75)	0.4051
	≥9 hours/day x No shift work	0.45 (-0.01, 0.91)	0.053794	0.68 (0.21, 1.14)	0.0046
	≥9 hours/day x Permanent night-shift	0.46 (-1.97, 2.90)	0.709417	0.49 (-1.99, 2.98)	0.6975
	6 hours/day x Day shift work	-0.32 (-0.92, 0.29)	0.304956	-0.48 (-1.10, 0.14)	0.1285
	6 hours/day x Mixed shift work	-0.66 (-1.41, 0.09)	0.086484	-0.82 (-1.59, -0.05)	0.0359
	6 hours/day x Night shift work	-1.20 (-2.48, 0.09)	0.06808	-1.79 (-3.11, -0.47)	0.0078
	6 hours/day x No shift work	-0.41 (-0.64, -0.19)	0.000357	-0.38 (-0.61, -0.15)	0.0014
	6 hours/day x Permanent night-shift	-0.16 (-1.21, 0.90)	0.771748	-0.45 (-1.53, 0.63)	0.4154
	7 hours/day x No shift work	Reference group (β-estimate = 0)			
	7 hours/day x Day shift work	-0.66 (-1.12, -0.21)	0.003999	-0.77 (-1.24, -0.31)	0.0012
	7 hours/day x Mixed shift work	-0.003 (-0.58, 0.58)	0.992315	-0.21 (-0.80, 0.39)	0.5014
	7 hours/day x Night shift work	-0.45 (-1.55, 0.65)	0.421739	-0.75 (-1.88, 0.38)	0.195
	7 hours/day x Permanent night-shift	-0.01 (-0.94, 0.92)	0.98664	-0.48 (-1.45, 0.48)	0.3256
	8 hours/day x Day shift work	-0.38 (-0.95, 0.19)	0.195288	-0.16 (-0.75, 0.42)	0.5879
	8 hours/day x Mixed shift work	-0.02 (-0.81, 0.77)	0.95864	0.29 (-0.52, 1.10)	0.478
	8 hours/day x Night shift work	-0.41 (-1.80, 0.98)	0.563461	-0.60 (-2.03, 0.82)	0.4068
	8 hours/day x No shift work	0.35 (0.15, 0.55)	0.000707	0.35 (0.14, 0.56)	0.001
	8 hours/day x Permanent night-shift	1.07 (-0.13, 2.28)	0.080192	0.65 (-0.59, 1.88)	0.305
	INTERACTION	β (95% CI)	p-value	β (95% CI)	p-value
Diast	≤5 hours/day x Day shift work	0.15 (-0.57, 0.87)	0.691	0.37 (-0.35, 1.10)	0.312

	≤5 hours/day x Mixed shift work	0.09 (-0.77, 0.96)	0.832	0.30 (-0.59, 1.18)	0.513
	≤5 hours/day x Night shift work	0.88 (-0.64, 2.39)	0.256	1.01 (-0.49, 2.51)	0.188
	≤5 hours/day x No shift work	0.12 (-0.15, 0.39)	0.387	0.16 (-0.12, 0.43)	0.267
	≤5 hours/day x Permanent night-shift	0.83 (-0.19, 1.86)	0.112	1.00 (-0.04, 2.04)	0.0603
	≥9 hours/day x Day shift work	0.21 (-0.55, 0.98)	0.583	0.07 (-0.72, 0.85)	0.867
	≥9 hours/day x Mixed shift work	-0.53 (-1.57, 0.51)	0.318	-0.35 (-1.41, 0.70)	0.513
	≥9 hours/day x Night shift work	0.48 (-1.28, 2.24)	0.594	0.05 (-1.75, 1.85)	0.957
	≥9 hours/day x No shift work	0.61 (0.34, 0.88)	9.19E-06	0.72 (0.44, 0.99)	3E-07
	≥9 hours/day x Permanent night-shift	-0.04 (-1.47, 1.39)	0.959	0.16 (-1.30, 1.62)	0.832
	6 hours/day x Day shift work	0.02 (-0.34, 0.37)	0.921	-0.08 (-0.44, 0.29)	0.681
	6 hours/day x Mixed shift work	-0.27 (-0.71, 0.17)	0.233	-0.28 (-0.73, 0.18)	0.23
	6 hours/day x Night shift work	-0.61 (-1.36, 0.15)	0.116	-0.74 (-1.51, 0.04)	0.062
	6 hours/day x No shift work	-0.09 (-0.22, 0.04)	0.178	-0.06 (-0.19, 0.08)	0.427
	6 hours/day x Permanent night-shift	0.28 (-0.34, 0.90)	0.379	0.22 (-0.41, 0.86)	0.49
	7 hours/day x No shift work	Reference group (β-estimate = 0)			
	7 hours/day x Day shift work	-0.33 (-0.60, -0.07)	0.0144	-0.37 (-0.64, -0.10)	0.0079
	7 hours/day x Mixed shift work	0.15 (-0.19, 0.49)	0.376	0.11 (-0.24, 0.47)	0.526
	7 hours/day x Night shift work	-0.07 (-0.71, 0.58)	0.84	-0.34 (-1.01, 0.32)	0.309
	7 hours/day x Permanent night-shift	0.33 (-0.22, 0.87)	0.238	0.11 (-0.45, 0.68)	0.696
	8 hours/day x Day shift work	0.06 (-0.28, 0.39)	0.75	0.16 (-0.18, 0.51)	0.357
	8 hours/day x Mixed shift work	0.18 (-0.29, 0.64)	0.457	0.33 (-0.14, 0.81)	0.171
	8 hours/day x Night shift work	-0.12 (-0.93, 0.69)	0.776	-0.05 (-0.88, 0.78)	0.906
	8 hours/day x No shift work	0.27 (0.15, 0.39)	0.000012	0.30 (0.18, 0.43)	1E-06
	8 hours/day x Permanent night-shift	0.27 (-0.43, 0.97)	0.452	-0.01 (-0.74, 0.71)	0.975

Supplementary Table S11. Pearson's correlation coefficients (r) values generated for associations between subpopulations of white blood cell counts, CRP levels, sleep length, sleep quality, shift work, SBP, DBP and key covariates including sex, age and BMI.

		**Blood pressure**		**Markers of systemic inflammation**								
		SBP	DBP	NeutPct	MonoCt	MonoPct	LymphCt	LymphPct	BasoPct	EosinoPct	LeukCt	CRP_IRN
Covariates	Age	0.3644	0.1189	-0.0007	0.0961	0.0791	0.005	-0.0213	-0.0139	-0.0009	0.0351	0.1348
	Sex	0.1698	0.1974	0.0406	0.233	0.2516	-0.1096	-0.1413	-0.0364	0.1026	0.0147	-0.0199
	BMI	0.239	0.334	-0.0067	0.1523	0.0133	0.1593	-0.0025	-0.0107	0.0647	0.1968	0.4268
BP	SBP	1	0.7461	0.0497	0.1315	0.0532	0.0372	-0.0663	-0.022	-0.015	0.1186	0.1725
	DBP	0.7461	1	0.0324	0.1242	0.0482	0.0505	-0.0487	-0.0227	0.0076	0.1138	0.1861
Sleep length	sleepCat_<=5	0.0152	0.0169	0.0132	0.0093	-0.0113	0.0118	-0.0115	-0.0001	-0.0059	0.0274	0.0374
	sleepCat_6	-0.0014	0.0093	0.0003	0.0068	0.0009	0.0065	-0.0008	0.0006	0.0027	0.0085	0.0134
	sleepCat_7	-0.0389	-0.0268	-0.0166	-0.0185	0.011	-0.0181	0.0147	-0.0011	0.009	-0.0397	-0.0551
	sleepCat_8	0.0127	-0.0028	0.002	-0.0018	-0.0045	0.0015	-0.0001	-0.0001	-0.004	0.0029	0.0023
	sleepCat_>=9	0.0414	0.0281	0.0167	0.0204	-0.0049	0.0122	-0.0171	0.0015	-0.0092	0.035	0.0497
Sleep quality	sleepQual_0-1	0.0528	0.0568	0.0063	0.0508	0.0204	0.0229	-0.017	0.0007	0.0188	0.0467	0.0833
	sleepQual_2-3	0.0145	0.0117	-0.0019	-0.0006	-0.0064	0.0084	0.0044	-0.0011	-0.0042	0.0064	0.0164
	sleepQual_4-5	-0.0592	-0.0592	-0.0029	-0.0403	-0.009	-0.0281	0.0086	0.0008	-0.0103	-0.0451	-0.0861
Shift work	shiftnight1_noshift	-0.0101	-0.0266	-0.0231	-0.0254	-0.002	-0.004	0.0275	0.0009	-0.0059	-0.0324	-0.0492
	shiftnight1_DayShift	0.0024	0.0063	0.0136	0.0032	-0.0088	0.0024	-0.0119	0.0005	-0.0019	0.0147	0.0285
	shiftnight1_MixedShift	0.0065	0.0203	0.0075	0.0214	0.0066	0.0085	-0.0113	0.0005	0.008	0.0216	0.0223
	shiftnight1_NightShift	0.0013	0.01	0.0099	0.0118	0.0066	-0.0042	-0.0138	-0.001	0.0017	0.0087	0.0179
	shiftnight1_PermanentNight	0.0108	0.0185	0.0142	0.018	0.0068	-0.0031	-0.02	-0.003	0.0056	0.0167	0.0249

Supplementary Table S12. β-estimate ± 95% CI for the association between each circadian rhythm-disrupting behaviour and BP following adjustments for CRP alone (CRP adj model) and CRP and BMI together (BMI and CRP adj model).

**SLEEP LENGTH AND BP**					
		**CRP adj model**		**BMI and CRP adj model**	
	**Sleep length**	**β (95% CI)**	**p-value**	**β (95% CI)**	**p-value**
**Systolic BP (mmHg)**	≤5	0.84 (0.51, 1.17)	0.00000053	0.28 (-0.05, 0.6)	0.0945
	6	0.14 (-0.05, 0.32)	0.144	-0.20 (-0.38, -0.02)	0.0285
	7	Reference group (β-estimate = 0)			
	8	0.20 (0.04, 0.36)	0.0158	0.24 (0.08, 0.4)	0.00254
	≥9	0.62 (0.34, 0.89)	0.0000125	0.39 (0.12, 0.67)	0.00463
**Diastolic BP (mmHg)**	≤5	0.82 (0.63, 1.01)	2.45E-17	0.33 (0.14, 0.51)	0.000458
	6	0.31 (0.2, 0.41)	1.49E-08	0.02 (-0.08, 0.12)	0.729
	7	Reference group (β-estimate = 0)			
	8	0.23 (0.14, 0.32)	0.000000826	0.27 (0.18, 0.36)	3.04E-09
	≥9	0.71 (0.55, 0.87)	2.49E-18	0.51 (0.36, 0.66)	6.74E-11
**SLEEP QUALITY AND BP**					
	**Sleep quality score**	**β (95% CI)**	**p-value**	**β (95% CI)**	**p-value**
**Systolic BP (mmHg)**	4 to 5	Reference group (β-estimate = 0)			
	2 to 3	0.52 (0.35, 0.69)	3.76E-09	0.07 (-0.10, 0.24)	0.43
	0 to 1	0.97 (0.71, 1.22)	9.98E-14	-0.23 (-0.49, 0.02)	0.0681
**Diastolic BP (mmHg)**	4 to 5	Reference group (β-estimate = 0)			
	2 to 3	0.69 (0.59, 0.79)	9.3E-42	0.30 (0.21, 0.40)	6.64E-10
	0 to 1	1.28 (1.13, 1.43)	2.54E-66	0.25 (0.11, 0.40)	0.000422
**SHIFT WORK AND BP**					
	**Shift work**	**β (95% CI)**	**p-value**	**β (95% CI)**	**p-value**
**Systolic BP (mmHg)**	No shift work	Reference group (β-estimate = 0)			
	Day-shift	-0.23 (-0.54, 0.08)	0.143	-0.50 (-0.81, -0.20)	0.00114
	Mixed-shift	0.15 (-0.25, 0.55)	0.451	-0.35 (-0.74, 0.04)	0.0787
	Night-shift	-0.52 (-1.23, 0.19)	0.149	-0.95 (-1.64, -0.25)	0.00732
	Permanent night-shift	0.45 (-0.14, 1.04)	0.135	-0.09 (-0.67, 0.48)	0.75
**Diastolic BP (mmHg)**	No shift work	Reference group (β-estimate = 0)			

	Day-shift	0.03 (-0.16, 0.21)	0.771	-0.21 (-0.39, -0.03)	0.0201
	Mixed-shift	0.40 (0.16, 0.64)	0.00111	-0.04 (-0.27, 0.19)	0.713
	Night-shift	0.02 (-0.4, 0.44)	0.93	-0.35 (-0.75, 0.06)	0.0965
	Permanent night-shift	0.55 (0.2, 0.9)	0.0022	0.10 (-0.24, 0.43)	0.582

Supplementary Table S13. β-estimate ± 95% CI for the association between sleep length and BP when stratified by low, medium and high levels of CRP and lymphocyte counts.

RELATIONSHIP BETWEEN SLEEP LENGTH AND BP: STRATIFIED BY CRP LEVELS AND LYMPHOCYTE COUNTS										
			CRP LEVELS				LYMPHOCYTE COUNT			
			Baseline model		BMI adj model		Baseline model		BMI adj model	
		Sleep length	β (95% CI)	p-value	β (95% CI)	p-value	β (95% CI)	p-value	β (95% CI)	p-value
**Systolic BP (mmHg)**	**Low**	≤5	0.89 (0.36, 1.42)	0.0011	0.44 (-0.09, 0.96)	0.103	1.72 (1.15, 2.29)	4E-09	0.97 (0.41, 1.53)	0.0007
		6	0.03 (-0.25, 0.30)	0.859	-0.27 (-0.54, 0.01)	0.0552	0.46 (0.14, 0.78)	0.0044	0.01 (-0.30, 0.32)	0.953
		7	Reference group (β-estimate = 0)							
		8	0.34 (0.1, 0.58)	0.0052	0.34 (0.11, 0.58)	0.0042	0.44 (0.16, 0.71)	0.0018	0.45 (0.18, 0.71)	0.0012
		≥9	0.98 (0.53, 1.43)	2E-05	0.69 (0.24, 1.13)	0.0023	0.91 (0.43, 1.40)	0.0002	0.49 (0.02, 0.96)	0.0417
	**Medium**	≤5	1.17 (0.63, 1.7)	2E-05	0.56 (0.03, 1.09)	0.0397	0.98 (0.40, 1.55)	0.0008	0.27 (-0.29, 0.83)	0.34
		6	0.32 (0.02, 0.62)	0.0394	-0.08 (-0.38, 0.21)	0.581	0.23 (-0.09, 0.54)	0.155	-0.19 (-0.50, 0.12)	0.224
		7	Reference group (β-estimate = 0)							
		8	0.33 (0.06, 0.59)	0.0148	0.33 (0.07, 0.59)	0.0126	0.20 (-0.07, 0.47)	0.147	0.20 (-0.06, 0.47)	0.136
		≥9	0.75 (0.3, 1.19)	0.0011	0.46 (0.02, 0.91)	0.039	1.20 (0.72, 1.67)	9E-07	0.68 (0.22, 1.15)	0.0042
	**High**	≤5	0.29 (-0.39, 0.96)	0.409	-0.35 (-1.02, 0.31)	0.3	0.73 (0.17, 1.29)	0.011	-0.18 (-0.73, 0.37)	0.511
		6	0.06 (-0.36, 0.48)	0.779	-0.30 (-0.71, 0.12)	0.158	0.03 (-0.29, 0.35)	0.859	-0.41 (-0.73, -0.09)	0.0109
		7	Reference group (β-estimate = 0)							
		8	-0.27 (-0.64, 0.10)	0.158	-0.15 (-0.52, 0.21)	0.416	0.19 (-0.10, 0.47)	0.197	0.19 (-0.08, 0.47)	0.167
		≥9	0.10 (-0.48, 0.67)	0.746	-0.12 (-0.69, 0.44)	0.669	0.58 (0.10, 1.05)	0.0175	0.21 (-0.26, 0.68)	0.377
**Diastolic BP (mmHg)**	**Low**	≤5	0.89 (0.58, 1.20)	2E-08	0.51 (0.22, 1.81)	0.0007	1.30 (0.97, 1.64)	2E-14	0.71 (0.39, 1.02)	1E-05
		6	0.29 (0.13, 0.45)	0.0004	0.06 (-0.10, 0.21)	0.467	0.41 (0.23, 0.60)	1E-05	0.07 (-0.11, 0.24)	0.455
		7	Reference group (β-estimate = 0)							
		8	0.31 (0.18, 0.45)	9E-06	0.32 (0.19, 0.45)	2E-06	0.32 (0.16, 0.48)	9E-05	0.34 (0.18, 0.49)	2E-05
		≥9	1.07 (0.80, 1.33)	1E-15	0.83 (0.58, 1.08)	1E-10	0.92 (0.64, 1.20)	1E-10	0.58 (0.32, 0.85)	2E-05
	**Medium**	≤5	0.82 (0.51, 1.12)	2E-07	0.28 (-0.02, 0.57)	0.068	0.83 (0.50, 1.16)	1E-06	0.23 (-0.08, 0.55)	0.149
		6	0.32 (0.15, 0.50)	0.0002	-0.03 (-0.19, 0.14)	0.772	0.40 (0.22, 0.59)	1E-05	0.07 (-0.11, 0.24)	0.445
		7	Reference group (β-estimate = 0)							
		8	0.30 (0.15, 0.45)	0.0001	0.30 (0.16, 0.45)	4E-05	0.26 (0.10, 0.42)	0.0012	0.26 (0.12, 0.41)	0.0005

		≥9	0.67 (0.41, 0.92)	3E-07	0.42 (0.17, 0.66)	0.0009	1.03 (0.75, 1.30)	3E-13	0.59 (0.32, 0.85)	1E-05
	**High**	≤5	0.69 (0.31, 1.08)	0.0004	0.11 (-0.26, 0.48)	0.546	0.93 (0.61, 1.25)	2E-08	0.14 (-0.17, 0.45)	0.37
		6	0.27 (0.03, 0.51)	0.0251	-0.04 (-0.27, 0.19)	0.714	0.37 (0.19, 0.56)	9E-05	-0.02 (-0.20, 0.15)	0.806
		7	Reference group (β-estimate = 0)							
		8	0.02 (-0.19, 0.24)	0.827	0.10 (-0.10, 0.30)	0.319	0.26 (0.10, 0.42)	0.0016	0.25 (0.10, 0.40)	0.0014
		≥9	0.52 (0.19, 0.84)	0.0022	0.28 (-0.04, 0.59)	0.0816	0.91 (0.64, 1.18)	7E-11	0.59 (0.33, 0.85)	9E-06

Supplementary Table S14. β-estimate ± 95% CI for the association between sleep length and BP when stratified by low, medium and high levels of monocytes and neutrophils.

RELATIONSHIP BETWEEN SLEEP LENGTH AND BP: STRATIFIED BY MONOCYTE AND NEUTROPHIL COUNT										
			MONOCYTE COUNT				NEUTROPHIL COUNT			
			Baseline model		BMI adj model		Baseline model		BMI adj model	
		Sleep length	β (95% CI)	p-value	β (95% CI)	p-value	β (95% CI)	p-value	β (95% CI)	p-value
Systolic BP (mmHg)	Low	≤5	1.53 (0.96, 1.21)	2E-07	0.68 (0.12, 1.23)	0.0177	1.72 (1.15, 2.29)	3E-09	1.03 (0.47, 1.58)	0.0003
		6	0.37 (0.06, 0.69)	0.0207	-0.05 (-0.36, 0.26)	0.746	0.32 (0.02, 0.63)	0.0385	-0.07 (-0.36, 0.23)	0.665
		7	Reference group (β-estimate = 0)							
		8	0.22 (-0.05, 0.49)	0.111	0.21 (-0.05, 0.48)	0.116	0.23 (-0.04, 0.49)	0.0918	0.25 (-0.01, 0.50)	0.0615
		≥9	0.66 (0.17, 1.14)	0.0078	0.32 (-0.16, 0.79)	0.191	1.17 (0.69, 1.66)	2E-06	0.77 (0.29, 1.25)	0.0015
	Medium	≤5	1.54 (0.97, 2.11)	1E-07	0.77 (0.21, 1.32)	0.0071	1.09 (0.51, 1.66)	0.0002	0.39 (-0.18, 0.95)	0.177
		6	0.22 (-0.09, 0.54)	0.166	-0.28 (-0.59, 0.03)	0.0746	0.05 (-0.27, 0.37)	0.753	-0.37 (-0.68, -0.06)	0.0206
		7	Reference group (β-estimate = 0)							
		8	0.33 (0.06, 0.60)	0.018	0.35 (0.08, 0.61)	0.0113	0.23 (-0.05, 0.50)	0.108	0.24 (-0.03, 0.50)	0.0867
		≥9	1.08 (0.59, 1.56)	1E-05	0.61 (0.13, 1.08)	0.0121	0.65 (0.17, 1.13)	0.0083	0.25 (-0.22, 0.71)	0.306
	High	≤5	0.29 (-0.28, 0.85)	0.318	-0.44 (-0.99, 0.12)	0.122	0.40 (-0.16, 0.95)	0.162	-0.44 (-0.99, 0.10)	0.111
		6	0.09 (-0.23, 0.42)	0.566	-0.28 (-0.60, 0.03)	0.0781	0.28 (-0.05, 0.61)	0.0974	-0.19 (-0.52, 0.13)	0.243
		7	Reference group (β-estimate = 0)							
		8	0.27 (-0.02, 0.55)	0.0648	0.28 (0.00, 0.55)	0.0521	0.22 (-0.07, 0.51)	0.131	0.23 (-0.05, 0.51)	0.106
		≥9	0.89 (0.42, 1.36)	0.0002	0.45 (-0.01, 0.91)	0.0536	0.46 (0.00, 0.93)	0.0521	0.09 (-0.37, 0.55)	0.71
Diastolic BP (mmHg)	Low	≤5	1.23 (0.90, 1.56)	3E-13	0.55 (0.24, 0.86)	0.0006	0.95 (0.62, 1.28)	2E-08	0.39 (0.07, 0.70)	0.0157
		6	0.50 (0.31, 0.68)	9E-08	0.15 (-0.02, 0.33)	0.0804	0.32 (0.14, 0.49)	0.0004	0.004 (-0.16, 0.17)	0.96
		7	Reference group (β-estimate = 0)							
		8	0.20 (0.04, 0.35)	0.0141	0.19 (0.04, 0.34)	0.0131	0.17 (0.02, 0.33)	0.0265	0.19 (0.04, 0.33)	0.0111
		≥9	0.74 (0.46, 1.02)	2E-07	0.45 (0.19, 0.72)	0.0009	0.99 (0.71, 1.27)	6E-12	0.66 (0.39, 0.93)	1E-06
	Medium	≤5	1.31 (0.98, 1.64)	7E-15	0.66 (0.35, 0.98)	3E-05	0.97 (0.64, 1.30)	1E-08	0.38 (0.06, 0.69)	0.02
		6	0.40 (0.22, 0.59)	2E-05	-0.01 (-0.19, 0.16)	0.881	0.42 (0.23, 0.60)	9E-06	0.07 (-0.11, 0.24)	0.447
		7	Reference group (β-estimate = 0)							
		8	0.33 (0.17, 0.48)	5E-05	0.34 (0.19, 0.49)	9E-06	0.34 (0.18, 0.50)	3E-05	0.35 (0.19, 0.50)	7E-06
		≥9	1.15 (0.87, 1.43)	9E-16	0.76 (0.49, 1.02)	2E-08	0.97 (0.69, 1.24)	8E-12	0.63 (0.37, 0.89)	3E-06
	High	≤5	0.50 (0.17, 0.83)	0.0028	-0.16 (-0.47, 0.16)	0.33	0.98 (0.65, 1.30)	3E-09	0.23 (-0.08, 0.54)	0.141

		6	0.27 (0.09, 0.46)	0.0042	-0.05 (-0.22, 0.13)	0.609	0.43 (0.24, 0.62)	1E-05	0.02 (-0.16, 0.20)	0.823
		7	Reference group (β-estimate = 0)							
		8	0.32 (0.15, 0.48)	0.0001	0.32 (0.16, 0.48)	6E-05	0.27 (0.10, 0.43)	0.0019	0.27 (0.11, 0.43)	0.0008
		≥9	0.93 (0.66, 1.20)	2E-11	0.55 (0.29, 0.80)	4E-05	0.72 (0.45, 0.99)	2E-07	0.37 (0.11, 0.63)	0.0053

Supplementary Table S15. β-estimate ± 95% CI for the association between sleep quality and BP when stratified by low, medium and high levels of CRP and lymphocyte counts.

SLEEP QUALITY AND BP: STRATIFIED BY BMI										
			**CRP LEVELS**				**LYMPHOCYTE COUNT**			
			**Baseline model**		**BMI adj model**		**Baseline model**		**BMI adj model**	
		**Sleep quality score**	**β (95% CI)**	**p-value**	**β (95% CI)**	**p-value**	**β (95% CI)**	**p-value**	**β (95% CI)**	**p-value**
**Systolic BP (mmHg)**	**Low**	4 to 5	Reference group (β-estimate = 0)							
		2 to 3	0.38 (0.14, 0.63)	0.0025	-0.06 (-0.31, 0.18)	0.618	0.82 (0.53, 1.12)	5E-08	0.19 (-0.10, 0.48)	0.205
		0 to 1	1.00 (0.59, 1.40)	2E-06	-0.17 (-0.57, 0.23)	0.405	1.61 (1.17, 2.06)	1E-12	0.01 (-0.43, 0.44)	0.977
	**Medium**	4 to 5	Reference group (β-estimate = 0)							
		2 to 3	0.61 (0.32, 0.90)	4E-05	0.13 (-0.16, 0.41)	0.381	0.86 (0.56, 1.15)	1E-08	0.16 (-0.13, 0.45)	0.271
		0 to 1	1.14 (0.73, 1.55)	6E-08	-0.05 (-0.46, 0.36)	0.814	1.70 (1.27, 2.13)	2E-14	0.04 (-0.39, 0.47)	0.846
	**High**	4 to 5	Reference group (β-estimate = 0)							
		2 to 3	0.91 (0.48, 1.34)	3E-05	0.29 (-0.13, 0.71)	0.174	0.64 (0.33, 0.94)	6E-05	-0.03 (-0.34, 0.27)	0.835
		0 to 1	1.07 (0.52, 1.63)	0.0002	-0.42 (-0.97, 0.13)	0.138	1.39 (0.96, 1.83)	4E-10	-0.35 (-0.78, 0.08)	0.112
**Diastolic BP (mmHg)**	**Low**	4 to 5	Reference group (β-estimate = 0)							
		2 to 3	0.61 (0.47, 0.75)	1E-16	0.26 (0.12, 0.40)	0.0002	0.80 (0.63, 0.97)	7E-20	0.31 (0.14, 0.47)	0.000245
		0 to 1	1.25 (1.01, 1.48)	5E-25	0.34 (0.11, 0.56)	0.0042	1.54 (1.28, 1.80)	1E-31	0.28 (0.03, 0.53)	0.0271
	**Medium**	4 to 5	Reference group (β-estimate = 0)							
		2 to 3	0.69 (0.52, 0.85)	4E-16	0.26 (0.10, 0.42)	0.0015	0.95 (0.78, 1.12)	5E-28	0.37 (0.21, 0.53)	6.33E-06
		0 to 1	1.30 (1.06, 1.53)	1E-27	0.24 (0.02, 0.47)	0.036	1.81 (1.56, 2.06)	3E-45	0.43 (0.19, 0.67)	0.000416
	**High**	4 to 5	Reference group (β-estimate = 0)							

		2 to 3	0.98 (0.73, 1.22)	4E-15	0.43 (0.20, 0.66)	0.0003	0.88 (0.70, 1.05)	6E-22	0.29 (0.12, 0.46)	0.000769
		0 to 1	1.53 (1.22, 1.85)	3E-21	0.21 (-0.10, 0.51)	0.183	1.74 (1.49, 1.99)	6E-42	0.22 (-0.02, 0.46)	0.076

Supplementary Table S16. β-estimate ± 95% CI for the association between sleep quality and BP when stratified by low, medium and high levels of monocytes and neutrophils.

SLEEP QUALITY AND BP: STRATIFIED BY BMI										
			MONOCYTE COUNT				NEUTROPHIL COUNT			
			Baseline model		BMI adj model		Baseline model		BMI adj model	
		Sleep quality score	β (95% CI)	p-value	β (95% CI)	p-value	β (95% CI)	p-value	β (95% CI)	p-value
Systolic BP (mmHg)	Low	4 to 5	Reference group (β-estimate = 0)							
		2 to 3	0.78 (0.49, 1.07)	1E-07	0.10 (-0.19, 0.38)	0.502	0.62 (0.34, 0.90)	2E-05	-0.02 (-0.29, 0.26)	0.902
		0 to 1	1.67 (1.22, 2.12)	3E-13	-0.11 (-0.55, 0.33)	0.632	1.32 (0.89, 1.75)	2E-09	-0.20 (-0.62, 0.22)	0.356
	Medium	4 to 5	Reference group (β-estimate = 0)							
		2 to 3	0.65 (0.35, 0.94)	2E-05	-0.05 (-0.34, 0.24)	0.741	0.72 (0.42, 1.01)	3E-06	0.12 (-0.18, 0.41)	0.437
		0 to 1	1.68 (1.24, 2.12)	6E-14	-0.01 (-0.44, 0.42)	0.952	1.88 (1.44, 2.31)	5E-17	0.27 (-0.16, 0.70)	0.225
	High	4 to 5	Reference group (β-estimate = 0)							
		2 to 3	0.86 (0.55, 1.18)	8E-08	0.26 (-0.05, 0.57)	0.103	0.99 (0.67, 1.30)	1E-09	0.26 (-0.05, 0.57)	0.104
		0 to 1	1.33 (0.89, 1.76)	2E-09	-0.22 (-0.65, 0.20)	0.303	1.41 (0.97, 1.86)	4E-10	-0.37 (-0.81, 0.07)	0.0996
Diastolic BP (mmHg)	Low	4 to 5	Reference group (β-estimate = 0)							
		2 to 3	0.84 (0.68, 1.01)	3E-23	0.29 (0.14, 0.45)	0.0003	0.76 (0.60, 0.92)	6E-20	0.24 (0.09, 0.40)	0.0024
		0 to 1	1.79 (1.53, 2.05)	7E-42	0.35 (0.10, 0.59)	0.006	1.48 (1.23, 1.73)	4E-31	0.24 (0.0, 0.48)	0.0484
	Medium	4 to 5	Reference group (β-estimate = 0)							
		2 to 3	0.88 (0.71, 1.05)	1E-23	0.30 (0.13, 0.46)	0.0004	0.88 (0.71, 1.06)	7E-24	0.38 (0.22, 0.55)	4.62E-06
		0 to 1	1.84 (1.58, 2.09)	7E-46	0.43 (0.19, 0.67)	0.0005	1.84 (1.59, 2.09)	1E-46	0.48 (0.24, 0.72)	0.000107
	High	4 to 5	Reference group (β-estimate = 0)							

		2 to 3	0.89 (0.70, 1.07)	2E-21	0.37 (0.19, 0.54)	4E-05	0.99 (0.81, 1.18)	8E-26	0.36 (0.18, 0.53)	7.75E-05
		0 to 1	1.49 (1.24, 1.74)	3E-31	0.15 (-0.09, 0.39)	0.214	1.76 (1.50, 2.02)	8E-41	0.21 (-0.04, 0.46)	0.094

Supplementary Table S17. β-estimate ± 95% CI for the association between shift work and BP when stratified by low, medium and high levels of CRP and lymphocytes.

RELATIONSHIP BETWEEN SHIFT WORKAND BP: STRATIFIED BY BMI										
			**CRP LEVELS**				**LYMPHOCYTE COUNT**			
			**Baseline model**		**BMI adj model**		**Baseline model**		**BMI adj model**	
		**Shift work**	**β (95% CI)**	**p-value**	**β (95% CI)**	**p-value**	**β (95% CI)**	**p-value**	**β (95% CI)**	**p-value**
**Systolic BP (mmHg)**	**Low**	No shift work	Reference group (β-estimate = 0)							
		Day-shift	-0.28 (-0.75, 0.18)	0.23	-0.55 (-1.01, -0.10)	0.017	0.12 (-0.42, 0.65)	0.669	-0.19 (-0.71, 0.33)	0.47
		Mixed-shift	0.59 (-0.01, 1.20)	0.0547	0.04 (-0.55, 0.63)	0.893	0.46 (-0.23, 1.15)	0.192	-0.33 (-1.00, 0.34)	0.335
		Night-shift	-0.37 (-1.48, 0.74)	0.514	-0.84 (-1.92, 0.25)	0.131	-0.42 (-1.61, 0.77)	0.49	-1.10 (-2.25, 0.06)	0.0625
		Permanent night-shift	0.49 (-0.43, 1.40)	0.297	-0.27 (-1.16, 0.63)	0.56	1.52 (0.53, 2.51)	0.0026	0.55 (-0.41, 1.51)	0.26
	**Medium**	No shift work	Reference group (β-estimate = 0)							
		Day-shift	-0.35 (-0.87, 0.16)	0.178	-0.63 (-1.13, -0.12)	0.0151	-0.08 (-0.60, 0.45)	0.768	-0.69 (-1.20, -0.18)	0.0079
		Mixed-shift	-0.21 (-0.86, 0.45)	0.533	-0.70 (-1.34, -0.06)	0.0329	0.68 (0.0, 1.36)	0.0491	-0.21 (-0.87, 0.46)	0.543
		Night-shift	-1.06 (-2.20, 0.08)	0.0679	-1.65 (-2.77, -0.53)	0.0039	0.45 (-0.76, 1.66)	0.463	-0.58 (-1.76, 0.60)	0.333
		Permanent night-shift	1.19 (0.22, 2.17)	0.0166	0.62 (-0.34, 1.57)	0.208	0.53 (-0.47, 1.52)	0.299	-0.42 (-1.39, 0.54)	0.388
	**High**	No shift work	Reference group (β-estimate = 0)							
		Day-shift	0.18 (-0.53, 0.88)	0.628	-0.19 (-0.87, 0.50)	0.598	-0.02 (-0.57, 0.52)	0.93	-0.46 (-0.99, 0.08)	0.0941
		Mixed-shift	0.37 (-0.55, 1.28)	0.433	-0.42 (-1.31, 0.47)	0.35	0.04 (-0.66, 0.74)	0.908	-0.53 (-1.21, 0.15)	0.126
		Night-shift	0.58 (-0.99, 2.14)	0.47	0.18 (-1.34, 1.71)	0.813	-0.28 (-1.56, 1.01)	0.674	-0.95 (-2.19, 0.30)	0.138
		Permanent night-shift	-0.32 (-1.59, 0.95)	0.617	-0.84 (-2.08, 0.39)	0.181	0.69 (-0.39, 1.77)	0.21	-0.10 (-1.15, 0.95)	0.855
	**Low**	No shift work	Reference group (β-estimate = 0)							

**Diastolic BP (mmHg)**		Day-shift	0.004 (-0.28, 0.29)	0.975	-0.21 (-0.48, 0.06)	0.133	0.25 (-0.08, 0.57)	0.134	-0.01 (-0.32, 0.29)	0.935
		Mixed-shift	0.51 (0.15, 0.88)	0.0062	0.06 (-0.29, 0.41)	0.742	0.59 (0.16, 1.01)	0.00666	-0.08 (-0.48, 0.32)	0.696
		Night-shift	0.18 (-0.49, 0.85)	0.605	-0.20 (-0.84, 0.45)	0.545	0.24 (-0.49, 0.96)	0.522	-0.31 (-0.99, 0.38)	0.384
		Permanent night-shift	0.59 (0.04, 1.14)	0.0359	-0.03 (-0.56, 0.51)	0.923	1.41 (0.80, 2.01)	4.9E-06	0.60 (0.03, 1.17)	0.0377
	**Medium**	No shift work	Reference group (β-estimate = 0)							
		Day-shift	0.04 (-0.26, 0.35)	0.781	-0.22 (-0.51, 0.07)	0.142	0.33 (0.01, 0.65)	0.0418	-0.19 (-0.49, 0.11)	0.21
		Mixed-shift	0.36 (-0.03, 0.75)	0.0704	-0.10 (-0.47, 0.28)	0.616	0.81 (0.40, 1.23)	0.00011	0.05 (-0.33, 0.44)	0.785
		Night-shift	0.01 (-0.67, 0.69)	0.977	-0.51 (-1.17, 0.14)	0.126	0.82 (0.09, 1.55)	0.0286	-0.04 (-0.73, 0.65)	0.901
		Permanent night-shift	0.95 (0.36, 1.53)	0.0015	0.43 (-0.13, 0.99)	0.13	0.62 (0.02, 1.22)	0.0426	-0.17 (-0.74, 0.39)	0.548
	**High**	No shift work	Reference group (β-estimate = 0)							
		Day-shift	0.13 (-0.30, 0.55)	0.555	-0.19 (-0.59, 0.20)	0.339	0.09 (-0.24, 0.42)	0.59	-0.28 (-0.59, 0.03)	0.0734
		Mixed-shift	0.51 (-0.04, 1.06)	0.0668	-0.17 (-0.69, 0.34)	0.507	0.35 (-0.07, 0.77)	0.105	-0.15 (-0.55, 0.24)	0.447
		Night-shift	0.03 (-0.91, 0.96)	0.959	-0.32 (-1.19, 0.56)	0.482	0.13 (-0.64, 0.90)	0.746	-0.45 (-1.17, 0.28)	0.226
		Permanent night-shift	0.17 (-0.59, 0.92)	0.66	-0.25 (-0.96, 0.46)	0.494	0.63 (-0.02, 1.27)	0.0584	-0.03 (-0.64, 0.59)	0.937

Supplementary Table S18. β-estimate ± 95% CI for the association between shift work and BP when stratified by low, medium and high levels of monocytes and neutrophils.

RELATIONSHIP BETWEEN SLEEP LENGTH AND BP: STRATIFIED BY BMI										
			MONOCYTE COUNT				NEUTROPHIL COUNT			
			Baseline model		BMI adj model		Baseline model		BMI adj model	
		**Shift work**	**β (95% CI)**	**p-value**	**β (95% CI)**	**p-value**	**β (95% CI)**	**p-value**	**β (95% CI)**	**p-value**
**Systolic BP (mmHg)**	**Low**	No shift work	Reference group (β-estimate = 0)							
		Day-shift	-0.35 (-0.87, 0.17)	0.188	-0.72 (-1.22, -0.22)	0.0051	-0.40 (-0.91, 0.11)	0.126	-0.79 (-1.29, -0.30)	0.00176
		Mixed-shift	0.29 (-0.41, 0.99)	0.423	-0.37 (-1.04, 0.31)	0.292	-0.001 (-0.68, 0.68)	0.997	-0.68 (-1.34, -0.03)	0.0418
		Night-shift	-0.94 (-2.18, 0.30)	0.138	-1.96 (-3.16, -0.76)	0.0014	0.35 (-0.84, 1.54)	0.562	-0.31 (-1.47, 0.85)	0.603
		Permanent night-shift	1.18 (0.13, 2.24)	0.0281	0.16 (-0.86, 1.18)	0.759	1.08 (0.07, 2.09)	0.0366	0.28 (-0.71, 1.26)	0.58
	**Medium**	No shift work	Reference group (β-estimate = 0)							
		Day-shift	0.31 (-0.22, 0.83)	0.259	-0.17 (-0.69, 0.34)	0.514	0.08 (-0.47, 0.62)	0.781	-0.42 (-0.95, 0.11)	0.123
		Mixed-shift	-0.12 (-0.81, 0.57)	0.735	-1.03 (-1.70, -0.36)	0.0026	0.78 (0.08, 1.48)	0.0295	-0.003 (-0.68, 0.68)	0.994
		Night-shift	0.55 (-0.67, 1.76)	0.38	0.02 (-1.17, 1.20)	0.98	-0.98 (-2.23, 0.26)	0.122	-1.77 (-2.98, -0.55)	0.00427
		Permanent night-shift	0.94 (-0.07, 1.95)	0.0674	0.08 (-0.90, 1.06)	0.871	0.44 (-0.57, 1.45)	0.393	-0.40 (-1.38, 0.59)	0.429
	**High**	No shift work	Reference group (β-estimate = 0)							
		Day-shift	-0.03 (-0.59, 0.53)	0.913	-0.49 (-1.03, 0.05)	0.076	0.11 (-0.44, 0.66)	0.691	-0.26 (-0.79, 0.27)	0.339
		Mixed-shift	0.89 (0.20, 1.57)	0.0111	0.27 (-0.40, 0.93)	0.434	0.07 (-0.61, 0.76)	0.832	-0.59 (-1.25, 0.08)	0.0869
		Night-shift	-0.06 (-1.29, 1.16)	0.919	-0.80 (-1.99, 0.39)	0.186	0.04 (-1.19, 1.27)	0.951	-0.70 (-1.90, 0.50)	0.253

		Permanent night-shift	0.37 (-0.63, 1.37)	0.464	-0.26 (-1.23, 0.71)	0.597	0.79 (-0.23, 1.81)	0.13	0.003 (-0.99, 0.99)	0.996
**Diastolic BP (mmHg)**	**Low**	No shift work	Reference group (β-estimate = 0)							
		Day-shift	-0.05 (-0.36, 0.27)	0.763	-0.37 (-0.66, -0.07)	0.0159	-0.05 (-0.36, 0.26)	0.753	-0.39 (-0.68, -0.09)	0.0104
		Mixed-shift	0.36 (-0.07, 0.78)	0.0991	-0.21 (-0.61, 0.20)	0.315	0.42 (0.00, 0.83)	0.0481	-0.16 (-0.55, 0.23)	0.411
		Night-shift	-0.23 (-0.98, 0.53)	0.554	-1.03 (-1.74, -0.32)	0.0046	0.54 (-0.19, 1.26)	0.148	0.001 (-0.69, 0.69)	0.997
		Permanent night-shift	0.89 (0.25, 1.53)	0.0063	0.06 (-0.54, 0.66)	0.854	1.14 (0.52, 1.75)	0.0003	0.44 (-0.14, 1.03)	0.136
	**Medium**	No shift work	Reference group (β-estimate = 0)							
		Day-shift	0.39 (0.07, 0.71)	0.0179	-0.01 (-0.31, 0.29)	0.944	0.32 (-0.01, 0.64)	0.0587	-0.11 (-0.42, 0.20)	0.475
		Mixed-shift	0.43 (0.01, 0.85)	0.0426	-0.34 (-0.73, 0.05)	0.091	0.80 (0.38, 1.22)	0.0002	0.13 (-0.27, 0.52)	0.539
		Night-shift	0.77 (0.03, 1.50)	0.0407	0.31 (-0.38, 1.00)	0.379	0.03 (-0.72, 0.78)	0.947	-0.65 (-1.35, 0.06)	0.0734
		Permanent night-shift	0.96 (0.35, 1.57)	0.0019	0.24 (-0.33, 0.81)	0.415	0.55 (-0.06, 1.16)	0.0759	-0.15 (-0.73, 0.42)	0.604
	**High**	No shift work	Reference group (β-estimate = 0)							
		Day-shift	0.28 (-0.06, 0.62)	0.11	-0.14 (-0.46, 0.18)	0.403	0.28 (-0.05, 0.61)	0.099	-0.05 (-0.36, 0.26)	0.746
		Mixed-shift	0.88 (0.46, 1.29)	4E-05	0.33 (-0.06, 0.72)	0.101	0.35 (-0.07, 0.77)	0.105	-0.25 (-0.64, 0.15)	0.223
		Night-shift	0.45 (-0.29, 1.19)	0.235	-0.19 (-0.89, 0.51)	0.586	0.36 (-0.39, 1.11)	0.343	-0.27 (-0.97, 0.44)	0.456
		Permanent night-shift	0.58 (-0.02, 1.19)	0.059	0.05 (-0.52, 0.62)	0.871	0.66 (0.04, 1.28)	0.0357	0.01 (-0.57, 0.59)	0.977

Sleep duration was identified as either reported an average of 7 hours of sleep as healthy (score of 0) or unhealthy for all other responses (score of 1). Participants who considered themselves to be “definitely a morning person” or more of a morning than evening person” were classified as having a morning chronotype (score of 0) and those who responded with either “definitely an evening person” or “more of the evening than morning person” were classified as having an evening chronotype (score of 1). Individuals who never or rarely experience daytime sleepiness (narcolepsy), insomnia/sleeplessness or snoring received a 0 or received an additional score of 1 if experienced sometimes or usually for each sleep disturbance.

For employment status, individuals were regrouped into four categories in response to the question, "Which of the following describes your current situation?": “currently employed” (in paid employment or self-employed); “unemployed” (unable to work because of sickness or disability, unemployed, looking after home and/or family and sick); “retired” (retired and looking after home and/or family); “unpaid volunteer” (Supplementary Table S2). Education (qualification) was separated into whether participants attended college (Attended college or university) or not attended college (all other responses) in response to “Which of the following qualifications do you have?”. Smoking and alcohol status were classified based on whether responses were “never”, “current” or “previous” for these covariates. Alcohol frequency was restricted to “Daily/almost daily”, 1-2 per week, 3-4 per week, special occasions only or never to the question, “"About how often do you drink alcohol?". Physical activity was organised into categories, <600MET-mins/week (reference category), 600_to_<1200MET-mins/week or ≥1200MET-mins/week.

Supplementary Table S19. Common anti-depressants, anti-psychotics, anxiolytics and medications for sleep purposes as previously outlined (Lane et al., 2017).

MEDICATION	NAME(S)
**Sleep medications**	oxazepam, meprobamate, medazepam, bromazepam, lorazepam, clobazam, chlormezanone, temazepam, nitrazepam, lormetazepam, diazepam, zopiclone, triclofos, methyprylone, prazepam, triazolam, ketazolam, dichloralphenazone, clomethiazole, zaleplon, butobarbital.
**Anti-depressants**	amitriptyline, citalopram, fluoxetine, sertraline, venlafaxine, dosulepin, paroxetine, mirtazapine, escitalopram, trazodone, prozac, seroxat, cipralex, duloxetine, lofepramine, clomipramine, nortriptyline, imipramine, dothiepin, cipramil, amitriptyline, prothiaden, trimipramine, lustral, reboxetine, zispin, cymbalta, anafranil, doxepin, moclobemide, phenelzine, fluvoxamine, yentreve, triptafen, surmontil, tranylcypromine, allegron, edronax, molipaxin, mianserin, nardil, faverin, nefazodone, amitriptyline+chlordiazepoxide, isocarboxazid, manerix, maoi, sinequan, tranylcypromine+trifluoperazine, ludiomil, norval, tryptizol, and fluphenazine hydrochloride+nortriptyline
**Anti-psychotics**	prochlorperazine, olanzapine, quetiapine, risperidone, chlorpromazine, trifluoperazine, amisulpride, sulpiride, seroquel, haloperidol, aripiprazole, stelazine, depixol, flupentixol, clozapine, promazine, risperdal, modecate, fluanxol, flupenthixol, zyprexa, zuclopenthixol, clopixol, largactil, abilify, fluphenazine, haldol, serenace, clozaril, cpz, perphenazine, levomepromazine, pericyazine, dolmatil, fentazin, fluphenazine, benperidol, pimozide, zaponex, denzapine, neulactil, thioridazine, dozic, fluspirilene, panadeine, and sertindole
**Anxiolytics**	zopiclone, diazepam, temazepam, zolpidem, nitrazepam, lorazepam, hydroxyzine, zimovane, phenergan, promethazine, buspirone, atarax, oxazepam, loprazolam, chlordiazepoxide, lormetazepam, ucerax, stilnoct, diazepam, buspar, alprazolam, librium, xanax, meprate, dalmane, clomethiazole, meprobamate, welldorm, amitriptyline+chlordiazepoxide, flurazepam, heminevrin, medazepam, neulactil, sinequan, almazine, atensine, carisoma, chloractil, chloral, dichloralphenazone, dormonoct, methyprylone, mogadon, rohypnol, tryptizol

Supplementary Table S20. Table of ICD codes and Data-Fields.

**Data-Field**	**Data-Field code**
List of medication	20003
Neutrophils	30140
Monocytes	30130
Basophils	30160
Eosinophils	30150
Total leukocytes	30000
Neutrophil to leukocyte ratio (NLR)	30200
CRP levels	30710
Employment status	6142
Smoking status	20116
Alcohol drinker status intake	20117
Alcohol frequency	1558
Physical activity	22040
Sleep duration (length)	1160
chronotype	1180
Sleeplessness/Insomnia	1200
Snoring	1210
Daytime sleepiness/dozing	1220
Shift work status - “Does your work involve shift work?”	826
Shift work schedule - "Does your work involve night shifts?”.	3426
Education	6138
**Medical condition**	**ICD9/ICD10 code**
Diagnosis of hypertension	I10-I15, 401-405
History of sleep apnea	G473
History of depression	F32, F320, F321, F322, F323, F328, F329, F33, F330, F331, F332, F333, F334, F338, F339, F34, F340, F341, F348, F349, F38, F380, F381, F388, F39

Supplementary Table S21. Classification of shift work type for all currently employed participants at the time of recruitment

**Question 1: Does your work involve shift work?**	**Question 2: Does your job involve night shifts?**	**Shift work type**
Never/rarely	-	No shift work (reference category)
Sometimes	Never/rarely	Day shift
	Sometime	Mixed shift
	Usually	Night shift
	Always	Night shift
Usually	Never/rarely	Day shift
	Sometime	Mixed shift
	Usually	Night shift
	Always	Night shift
Always	Never/rarely	Day shift
	Sometime	Mixed shift
	Usually	Night shift
	Always	Permanent night shift

Supplementary Table S22. BP-lowering medications outlined by the American Heart Association (https://www.heart.org/en/).

ANTI-HYPERTENSIVES			
**CLASS**	**TYPE**	**GENERIC DRUG NAME**	**COMMON BRAND NAME**
**DIURETICS**	**Thiazide diuretics**	chlorthalidone	Hygroton
		chlorothiazide	Diuril
		hydrochlorothiazide	-
		indapamide	-
		metolazone	-
	**Potassium-sparing diuretics**	amiloride hydrochloride	-
		spironolactone	Aldactone
		eplerenone	Inspra
		triamterene	-
	**Loop diuretic**	furosemide	Lasix
		bumetanide	-
**BETA-BLOCKERS**		acebutolol	Sectral
		atenolol	Tenormin
		betaxolol	Kerlone
		bisoprolol fumarate	-
		carteolol	Cartrol
		metoprolol tartrate	-
		nadolol	Corgard
		penbutolol sulphate	-
		pindolol	Visken
		propranolol hydrochloride	Inderal
		solotol hydrochloride	Betapace
		timolol maleate	Blocadren
**CALCIUM CHANNEL BLOCKERS**		amlodipine	-
		felodipine	Plendil
		isradipine	-
		nicardipine	Cardene
		nifedipine	Adalat
		nisoldipine	-
		verapamil hydrochloride	Calan

**ANGIOTENSIN II RECEPTOR BLOCKERS (ARB)**	candesartan	-
	eprosartan mesylate	Teveten
	losartan potassium	Cozaar
	telmisartan	Micardis
	valsartan	Diovan
	captopril	Capoten
	enalapril maleate	-
	fosinopril	-
	lisinopril	Zestril
	moexipril	-
	perindopril	-
	quinapril	-
	ramipril	-
	trandolapril	-
**ALPHA-BLOCKERS**	doxazosin	Cardura
	prazosin	-
	terazosin	Hytrin
**ALPHA-2 RECEPTOR BLOCKERS**	methyldopa	N/A
**VASODILATORS**	hydralazine	Apresoline
	minoxidil	Loniten
**CENTRAL AGONISTS**	alpha methyldopa	Aldomet
	clonidine hydrochloride	Catapres
**PERIPHERAL ADRENERGIC RECEPTORS**	Guanethidine monosulphate	Ismelin
	Reserpine	Serpasil

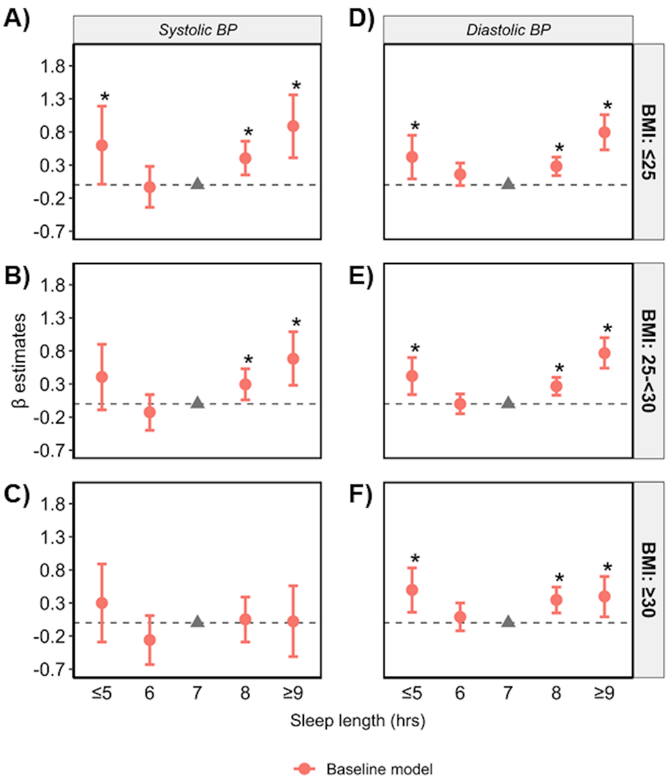

Supplementary Figure S1. Sleep length versus BP stratified by BMI. Relationship between sleep length and SBP (A, B, C) and DBP (D, E, F) after data is stratified by BMI groups, ≤25, 25-<30 and ≥30 kg/m^2, respectively. Baseline model: Adjusted for age, sex, smoking status, alcohol frequency, alcohol intake, education/qualification, employment status, history of depression, physical activity and Townsend deprivation index; BMI adj model: Baseline model plus further adjusting for BMI. Data are expressed as β-estimate ± 95% CI, which are presented as the centre circle and corresponding error bars, respectively. *, $p<0.05$ versus sleep length of 7hr denoted by the solid grey triangle. P-values are estimated using multivariate logistic regression.

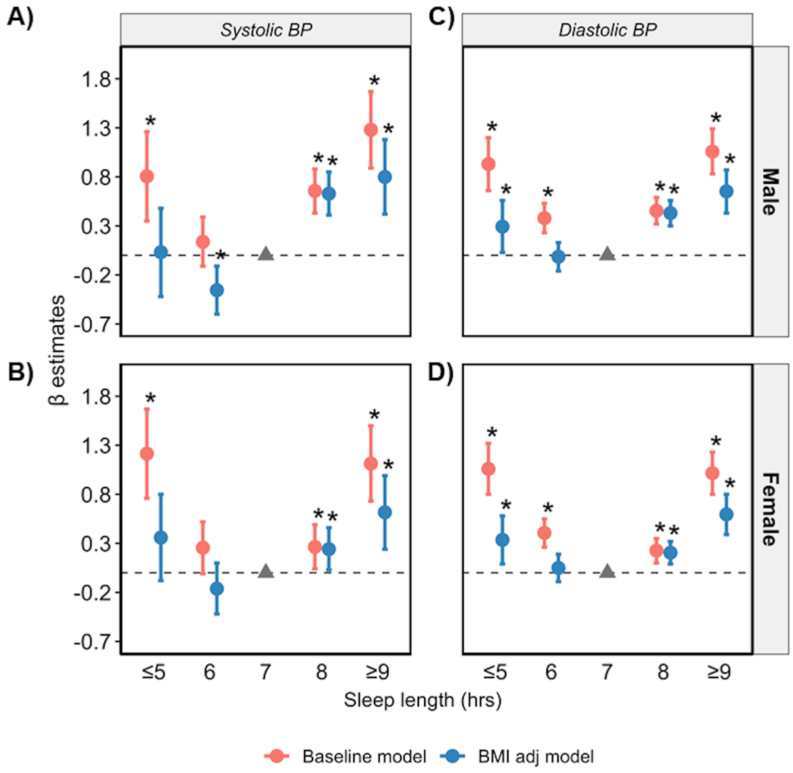

Supplementary Figure S2. Sleep length versus BP stratified by sex. Relationship between sleep length and SBP (A, B) and DBP (C, D) in males and females, respectively, following the effect of excluding (Baseline model, pink) and including (BMI adj model, blue) adjusting for BMI. Baseline model: Adjusted for age, sex, smoking status, alcohol frequency, alcohol intake, education/qualification, employment status, history of depression, physical activity, and Townsend deprivation index; BMI adj model: Baseline model and further adjusting for BMI. Data are expressed as β-estimate ± 95% CI, which are presented as the centre circle and corresponding error bars, respectively. *, $p<0.05$ versus sleep length of 7hr denoted by the solid grey triangle. P-values are estimated using multivariate logistic regression.

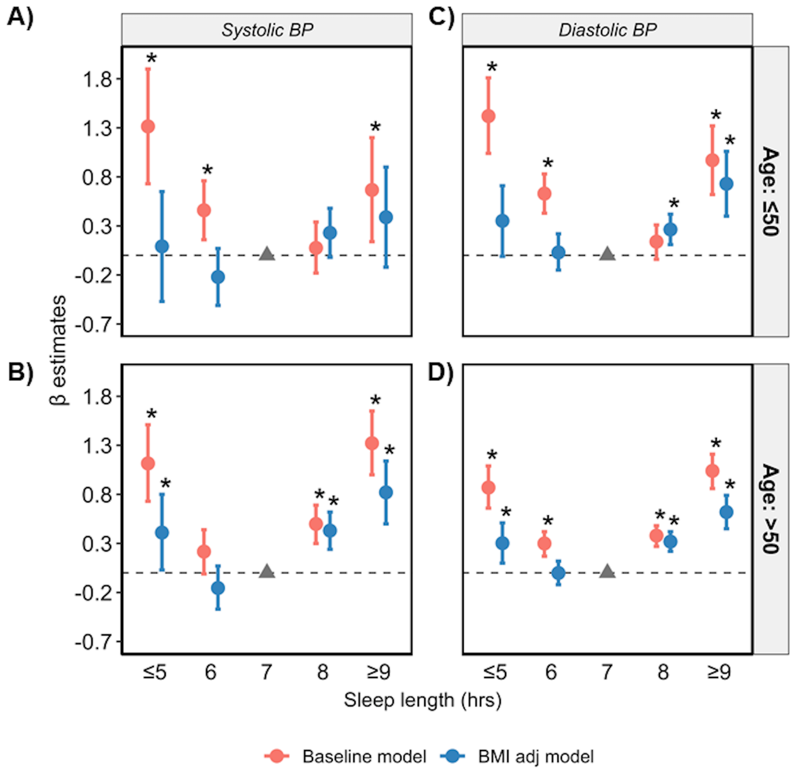

Supplementary Figure S3. Sleep length versus BP stratified by age. Relationship between sleep length and SBP (A, B) and DBP (C, D) after data is stratified by ages ≤50 or >50 years old, respectively, following the effect of excluding (Baseline model, pink) and including (BMI adj model, blue) adjusting for BMI. Baseline model: Adjusted for age, sex, smoking status, alcohol frequency, alcohol intake, education/qualification, employment status, history of depression, physical activity, and Townsend deprivation index; BMI adj model: Data are expressed as β-estimate ± 95% CI, which are presented as the centre circle and corresponding error bars, respectively. *, p<0.05 versus sleep length of 7hr denoted by the solid grey triangle. P-values are estimated using multivariate logistic regression. P-values are estimated using multivariate logistic regression.

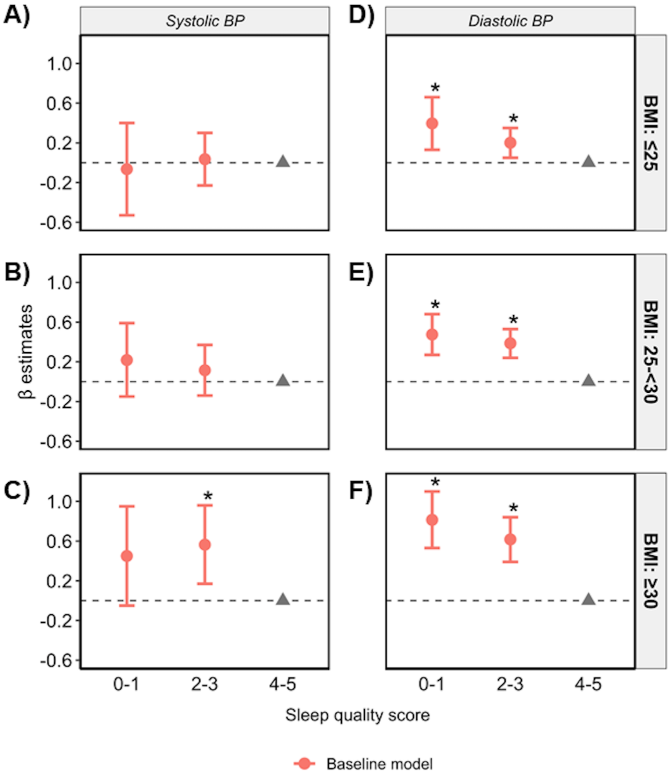

Supplementary Figure S4. Sleep quality versus BP stratified by BMI. Relationship between sleep quality and SBP (A, B, C) and DBP (D, E, F) after data is stratified by BMI groups, ≤25, 25-<30 and ≥30 kg/m^2, respectively. Baseline model: Adjusted for age, sex, smoking status, alcohol frequency, alcohol intake, education/qualification, employment status, history of depression, physical activity and Townsend deprivation index; BMI adj model: Baseline model plus further adjusting for BMI. Data are expressed as β-estimate ± 95% CI, which are presented as the centre circle and corresponding error bars, respectively. *, p<0.05 versus a sleep quality score of 4-5 denoted by the solid grey triangle. P-values are estimated using multivariate logistic regression.

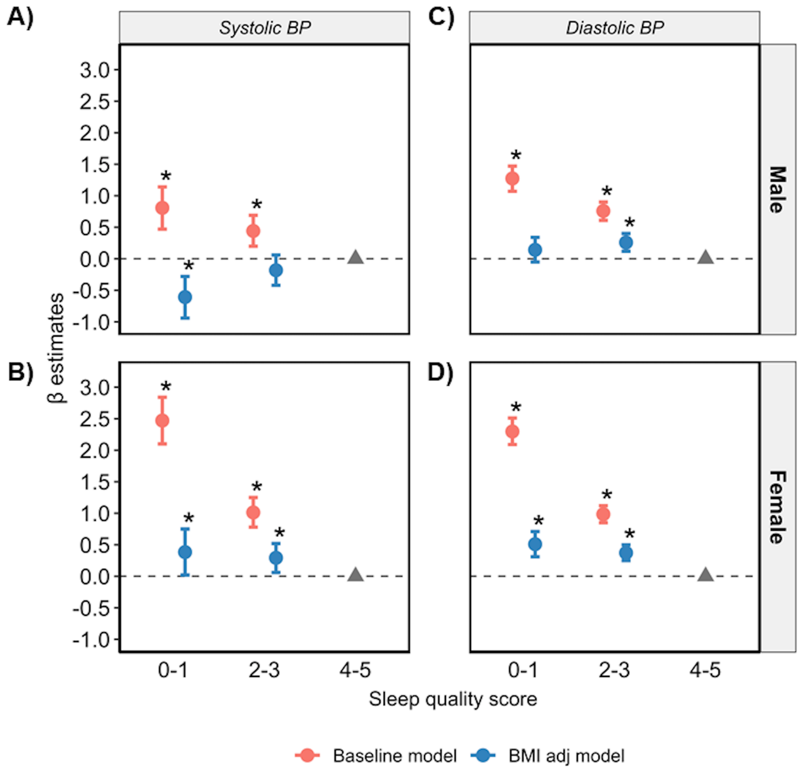

Supplementary Figure S5. Sleep quality versus BP stratified by sex. Relationship between sleep length and SBP (A, B) and DBP (C, D) in males and females, respectively, following the effect of excluding (Baseline model, pink) and including (BMI adj model, blue) adjusting for BMI. Baseline model: Adjusted for age, sex, smoking status, alcohol frequency, alcohol intake, education/qualification, employment status, history of depression, physical activity, and Townsend deprivation index; BMI adj model: Baseline model and further adjusting for BMI. Data are expressed as β-estimate ± 95% CI, which are presented as the centre circle and corresponding error bars, respectively. *, $p<0.05$ versus a sleep quality score of 4-5 denoted by the solid grey triangle. P-values are estimated using multivariate logistic regression.

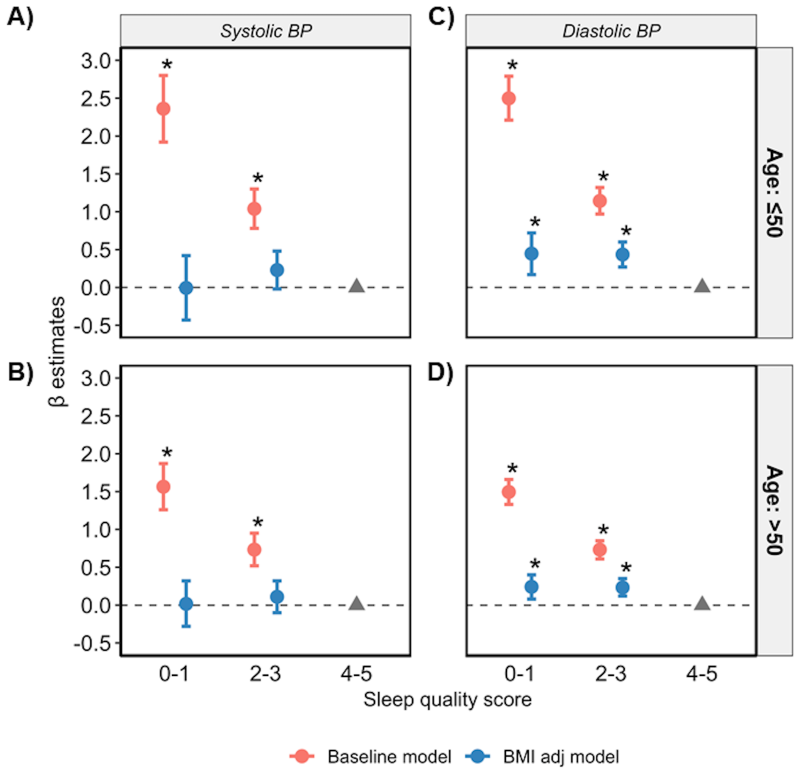

Supplementary Figure S6. Sleep quality versus BP stratified by age. Relationship between sleep quality and SBP (A, B) and DBP (C, D) after data is stratified by age ≤50 or >50 years old, respectively, following the effect of excluding (Baseline model, pink) and including (BMI adj model, blue) adjusting for BMI. Baseline model: Adjusted for age, sex, smoking status, alcohol frequency, alcohol intake, education/qualification, employment status, history of depression, physical activity, and Townsend deprivation index; BMI adj model: Baseline model and further adjusting for BMI. Data are expressed as β-estimate ± 95% CI, which are presented as the centre circle and corresponding error bars, respectively. *, $p<0.05$ versus a sleep quality score of 4-5 denoted by the solid grey triangle. P-values are estimated using multivariate logistic regression.

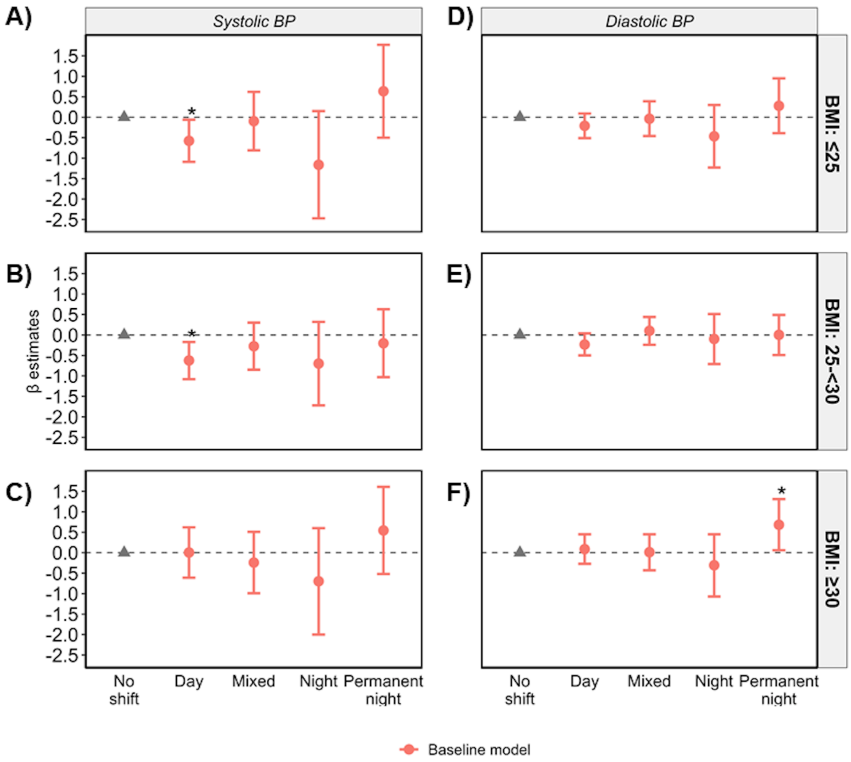

Supplementary Figure S7. Shift work versus BP stratified by BMI. Relationship between shift work and SBP (A, B, C) and DBP (D, E, F) after data is stratified by BMI groups, ≤25, 25-<30 and ≥30 kg/m^2, respectively. Baseline model: Adjusted for age, sex, smoking status, alcohol frequency, alcohol intake, education/qualification, employment status, history of depression, physical activity and Townsend deprivation index; BMI adj model: Baseline model plus further adjusting for BMI. Data are expressed as β-estimate ± 95% CI, which are presented as the centre circle and corresponding error bars, respectively. *, $p<0.05$ versus the no shift work group denoted by the solid grey triangle. P-values are estimated using multivariate logistic regression.

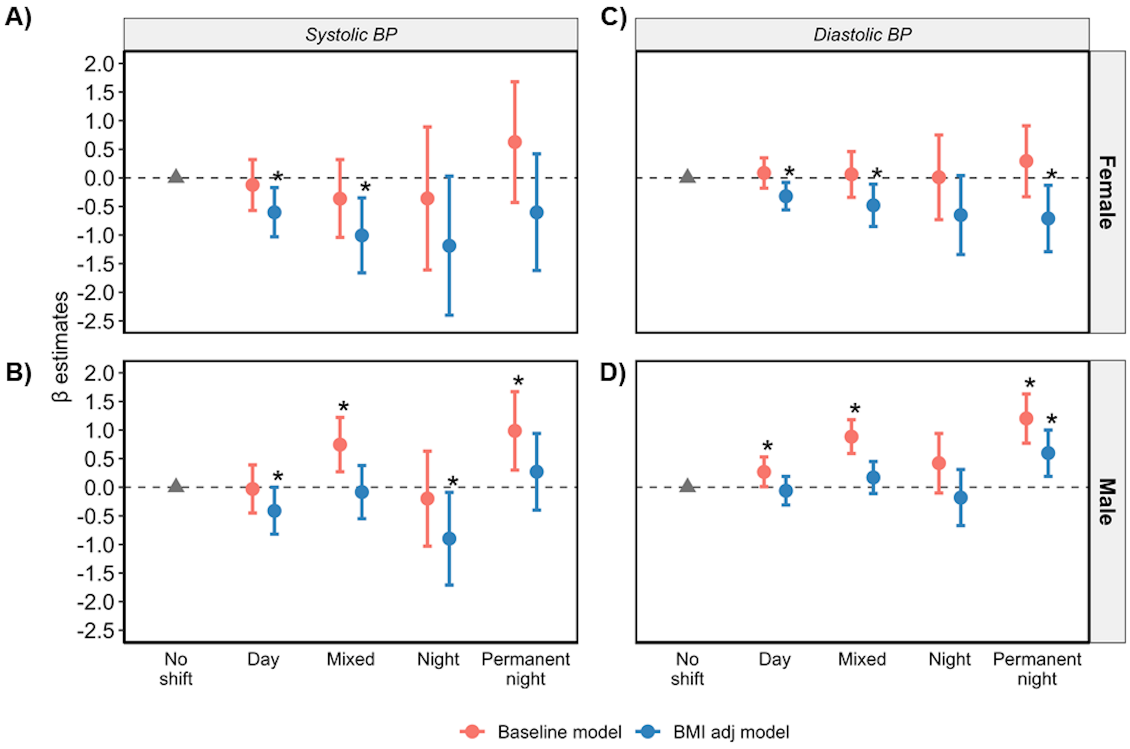

Supplementary Figure S8. Shift work versus BP stratified by sex. Relationship between shift work and SBP (A, B) and DBP (C, D) in males and females, respectively, following the effect of excluding (Baseline model, pink) and including (BMI adj model, blue) adjusting for BMI. Baseline model: Adjusted for age, sex, smoking status, alcohol frequency, alcohol intake, education/qualification, employment status, history of depression, physical activity, and Townsend deprivation index; BMI adj model: Baseline model and further adjusting for BMI. Data are expressed as β-estimate ± 95% CI, which are presented as the centre circle and corresponding error bars, respectively. *, $p<0.05$ versus the no shift work group denoted by the solid grey triangle. P-values are estimated using multivariate logistic regression.

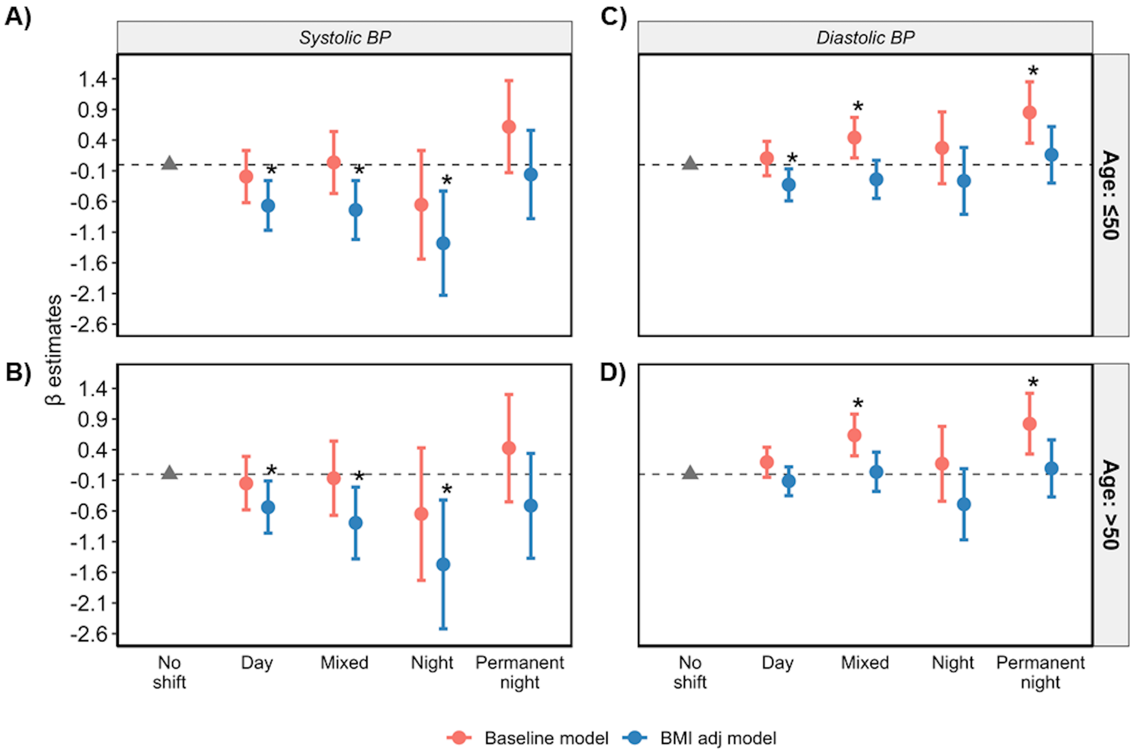

Supplementary Figure S9. Shift work versus BP stratified by age. Relationship between sleep quality and SBP (A, B) and DBP (C, D) after data is stratified by age ≤50 or >50 years old, respectively, following the effect of excluding (Baseline model, pink) and including (BMI adj model, blue) adjusting for BMI. Baseline model: Adjusted for age, sex, smoking status, alcohol frequency, alcohol intake, education/qualification, employment status, history of depression, physical activity, and Townsend deprivation index; BMI adj model: Baseline model and further adjusting for BMI. Data are expressed as β-estimate ± 95% CI, which are presented as the centre circle and corresponding error bars, respectively. *, $p<0.05$ versus the no shift work group denoted by the solid grey triangle. P-values are estimated using multivariate logistic regression.

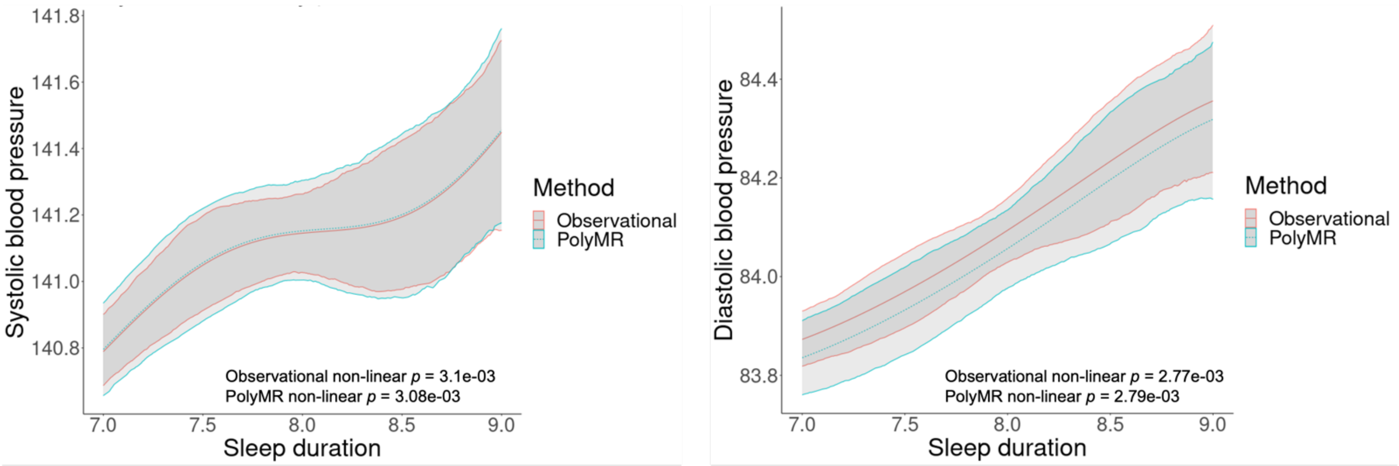

Supplementary Figure S10. PolyMR fitting 10th-degree polynomial between sleep duration and blood pressure. Outcomes (Y) were adjusted for covariates of sex, BMI, age, C-reactive protein levels, lymphocyte levels, monocyte levels, and neutrophils levels. p-values are estimated using PolyMR testing the null hypothesis that the relationships between exposure and outcome are linear.

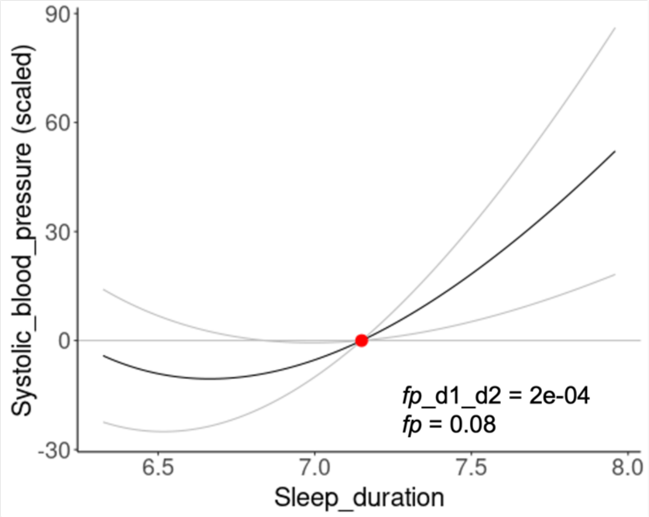

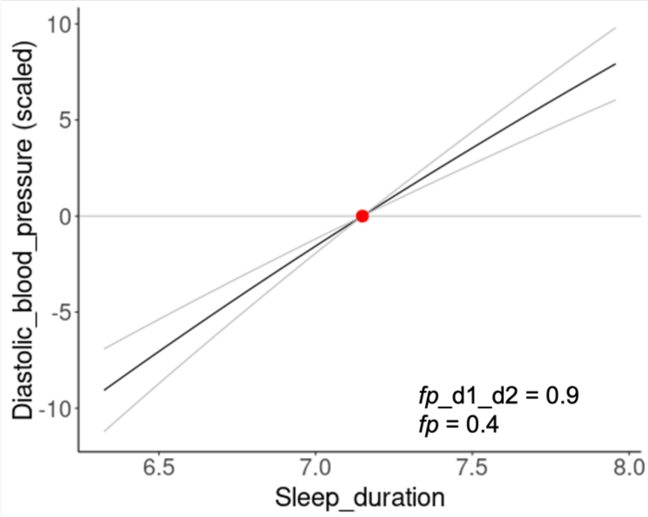

Supplementary Figure S11. SUMnlmr fitting 1- or 2-degree fractional polynomials sleep duration and blood pressure. fp_d1_d2: p-value of degree 1 or 2 fractional polynomials. fp: a low p-value indicates a preference for a non-linear fractional polynomial model compared with a linear model. p-values are estimated using SUMnlmr.

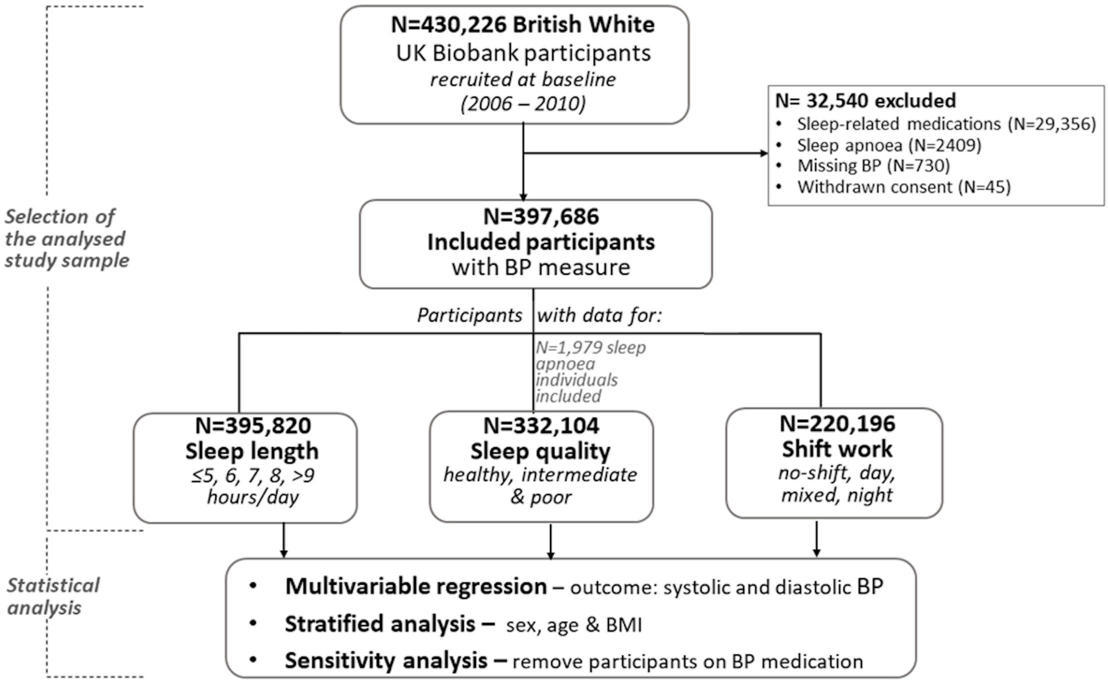

Supplementary Figure S12. Study flow chart. Classification of participants based on self-reported sleep length, assigned sleep quality score and self-reported shift work schedules.

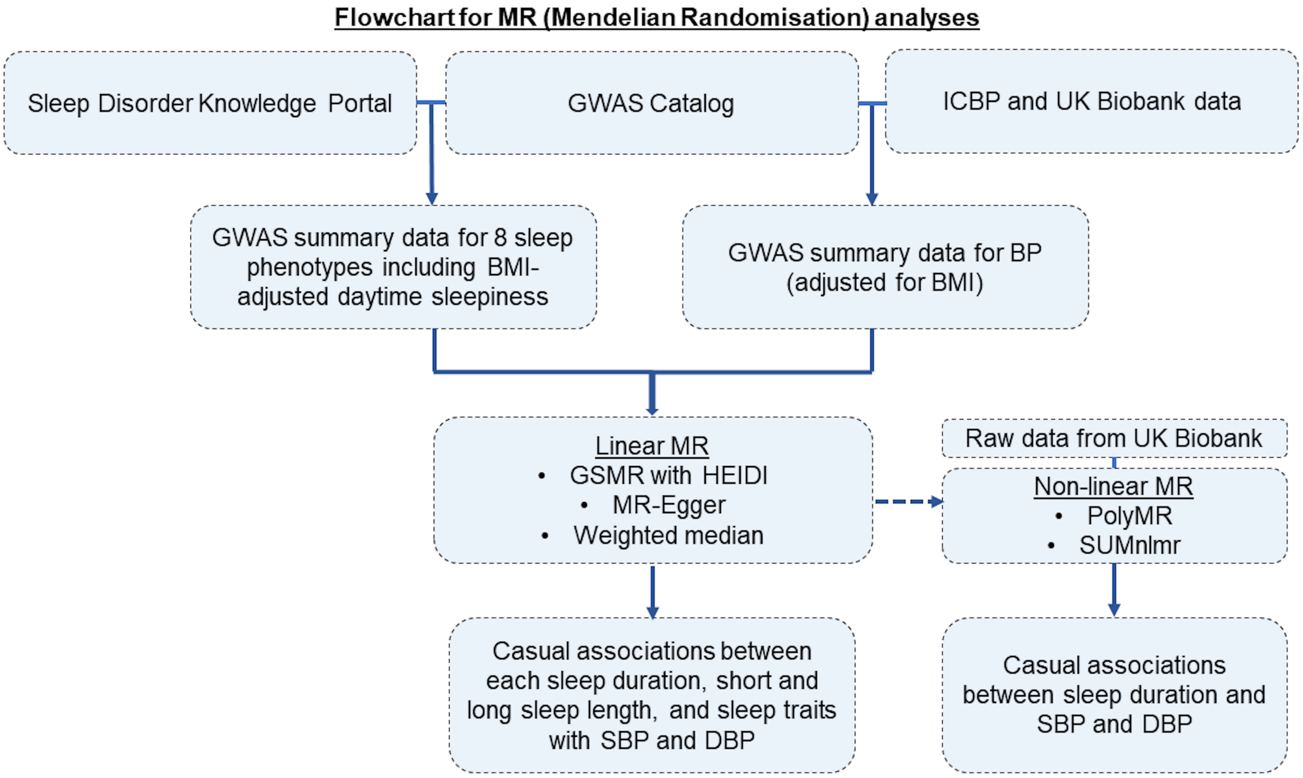

Supplementary Figure S13. Mendelian randomisation (MR) flow chart. GWAS-based MR summary data was used to perform linear and non-linear MR analyses to identify causal associations between sleep phenotypes and SBP/DBP.

STROBE-MR checklist of recommended items to address in reports of Mendelian randomization studies[1,2]

Item No.	Section	Checklist item	Page No.	Relevant text from manuscript
1	**TITLE and ABSTRACT**	Indicate Mendelian randomization (MR) as the study's design in the title and/or the abstract if that is a main purpose of the study	NA	MR is only used for validation
	**INTRODUCTION**			
2	**Background**	Explain the scientific background and rationale for the reported study. What is the exposure? Is a potential causal relationship between exposure and outcome plausible? Justify why MR is a helpful method to address the study question	3-4	MR analyses was motivated by observational association analyses between exposures and outcomes
3	**Objectives**	State specific objectives clearly, including pre-specified causal hypotheses (if any). State that MR is a method that, under specific assumptions, intends to estimate causal effects	4	To assess if sleep disruptions have causal associations with blood pressure
	**METHODS**			
4	**Study design and data sources**	Present key elements of the study design early in the article. Consider including a table listing sources of data for all phases of the study. For each data source contributing to the analysis, describe the following:	8-9, 18-20	Published GWAS summary statistics from sleep phenotypes(http://sleepdisordergenetics.org/) and from blood pressure (https://www.nature.com/articles/s41588-018-0205-x)
	a)	Setting: Describe the study design and the underlying population, if possible. Describe the setting, locations, and relevant dates, including periods of recruitment, exposure, follow-up, and data collection, when available.	8-9, 18-20	Consisted of individuals from ~1 million people with multiple ancestries, the raw data are detailed in http://sleepdisordergenetics.org/ and https://www.nature.com/articles/s41588-018-0205-x)
	b)	Participants: Give the eligibility criteria, and the sources and methods of selection of participants. Report the sample size, and whether any power or sample size calculations were carried out prior to the main analysis	8-9, 18-20	Consisted of individuals from ~1 million people with multiple ancestries, the raw data are detailed in http://sleepdisordergenetics.org/ and https://www.nature.com/articles/s41588-018-0205-x)
	c)	Describe measurement, quality control and selection of genetic variants	18-20	Variants with LD-r2 < 0.05 were used, F-stats for variants ranging from 34.2 to 51.2
	d)	For each exposure, outcome, and other relevant variables, describe methods of assessment and diagnostic criteria for diseases	18-20	Consisted of individuals from ~1 million people with multiple ancestries, the raw data are detailed in http://sleepdisordergenetics.org/ and https://www.nature.com/articles/s41588-018-0205-x)

		e)	Provide details of ethics committee approval and participant informed consent, if relevant	NA	
5	**Assumptions**		Explicitly state the three core IV assumptions for the main analysis (relevance, independence and exclusion restriction) as well assumptions for any additional or sensitivity analysis	20	Three established MR methods are used to ensure that 1) tested SNPs are associated with the exposure (p < 5e-8). 2) SNPs are not associated with confounder (e.g., BMI were adjusted) and 3) SNPs are expected to influence the blood pressure via sleep disruptions.
6	**Statistical methods: main analysis**		Describe statistical methods and statistics used	18-20	See sections “GWAS summary-based MR” and “Statistical analyses”
		a)	Describe how quantitative variables were handled in the analyses (i.e., scale, units, model)	18-19	See sections “GWAS summary-based MR”
		b)	Describe how genetic variants were handled in the analyses and, if applicable, how their weights were selected	18-19	See sections “GWAS summary-based MR”, e.g., Variants with LD-r2 < 0.05 were used, F-stats for variants ranging from 34.2 to 51.2
		c)	Describe the MR estimator (e.g. two-stage least squares, Wald ratio) and related statistics. Detail the included covariates and, in case of two-sample MR, whether the same covariate set was used for adjustment in the two samples	18-19	See sections “GWAS summary-based MR”
		d)	Explain how missing data were addressed	NA	
		e)	If applicable, indicate how multiple testing was addressed	20	FDR-adjusted p-values were used to declare significance of linear MR
7	**Assessment of assumptions**		Describe any methods or prior knowledge used to assess the assumptions or justify their validity	18	The motivation for conducting MR is to validate the association analyses conducted described earlier.
8	**Sensitivity analyses and additional analyses**		Describe any sensitivity analyses or additional analyses performed (e.g. comparison of effect estimates from different approaches, independent replication, bias analytic techniques, validation of instruments, simulations)	20-21	We used tested non-linear MR using PolyMR and SUMnlmr to conduct additional validation analyses
9	**Software and pre-registration**				
		a)	Name statistical software and package(s), including version and settings used	18-21	See sections “GWAS summary-based MR”
		b)	State whether the study protocol and details were pre-registered (as well as when and where)	NA	

RESULTS

10	**Descriptive data**				
		a)	Report the numbers of individuals at each stage of included studies and reasons for exclusion. Consider use of a flow diagram	18-21	See sections “GWAS summary-based MR”
		b)	Report summary statistics for phenotypic exposure(s), outcome(s), and other relevant variables (e.g. means, SDs, proportions)	18-21	See sections “GWAS summary-based MR”
		c)	If the data sources include meta-analyses of previous studies, provide the assessments of heterogeneity across these studies	18-21	See sections “GWAS summary-based MR”
		d)	For two-sample MR:<br>i. Provide justification of the similarity of the genetic variant-exposure associations between the exposure and outcome samples<br>ii. Provide information on the number of individuals who overlap between the exposure and outcome studies	18-21	See sections “GWAS summary-based MR”
11	**Main results**				
		a)	Report the associations between genetic variant and exposure, and between genetic variant and outcome, preferably on an interpretable scale	8-9	See section “Short and long sleep lengths, chronotype daytime sleepiness and daytime napping are causally linked to BP”
		b)	Report MR estimates of the relationship between exposure and outcome, and the measures of uncertainty from the MR analysis, on an interpretable scale, such as odds ratio or relative risk per SD difference	8-9	See section “Short and long sleep lengths, chronotype daytime sleepiness and daytime napping are causally linked to BP”
		c)	If relevant, consider translating estimates of relative risk into absolute risk for a meaningful time period	NA	
		d)	Consider plots to visualize results (e.g. forest plot, scatterplot of associations between genetic variants and outcome versus between genetic variants and exposure)	8-9	See Table 2 and Figure 8 and supplementary Figure 10-11
12	**Assessment of assumptions**				
		a)	Report the assessment of the validity of the assumptions	18	The motivation for conducting MR is to validate the association analyses conducted described earlier.
		b)	Report any additional statistics (e.g., assessments of heterogeneity across genetic variants, such as I^2, Q statistic or E-value)	18-21	See sections “GWAS summary-based MR”
13	**Sensitivity analyses and additional analyses**			NA	

		a)	Report any sensitivity analyses to assess the robustness of the main results to violations of the assumptions	NA	
		b)	Report results from other sensitivity analyses or additional analyses	NA	
		c)	Report any assessment of direction of causal relationship (e.g., bidirectional MR)	NA	
		d)	When relevant, report and compare with estimates from non-MR analyses	8-9	See section “Short and long sleep lengths, chronotype daytime sleepiness and daytime napping are causally linked to BP”
		e)	Consider additional plots to visualize results (e.g., leave-one-out analyses)	8-9	See Figure 8 and supplementary Figure 10-11
	**DISCUSSION**				
14	**Key results**		Summarize key results with reference to study objectives	10-11	See the 1st and 3rd paragraphs of Discussion
15	**Limitations**		Discuss limitations of the study, taking into account the validity of the IV assumptions, other sources of potential bias, and imprecision. Discuss both direction and magnitude of any potential bias and any efforts to address them	11	See the 3rd paragraphs of Discussion
16	**Interpretation**				
		a)	Meaning: Give a cautious overall interpretation of results in the context of their limitations and in comparison with other studies	10-11	See the 1st and 3rd paragraphs of Discussion
		b)	Mechanism: Discuss underlying biological mechanisms that could drive a potential causal relationship between the investigated exposure and the outcome, and whether the gene-environment equivalence assumption is reasonable. Use causal language carefully, clarifying that IV estimates may provide causal effects only under certain assumptions	10-11	See the 1st and 3rd paragraphs of Discussion
		c)	Clinical relevance: Discuss whether the results have clinical or public policy relevance, and to what extent they inform effect sizes of possible interventions	11	See the 3rd paragraphs of Discussion
17	**Generalizability**		Discuss the generalizability of the study results (a) to other populations, (b) across other exposure periods/timings, and (c) across other levels of exposure	NA	
	**OTHER INFORMATION**				
18	**Funding**		Describe sources of funding and the role of funders in the present study and, if applicable, sources of funding for the databases and original study or studies on which the present study is based	28	UK Biobank Resource (https://www.ukbiobank.ac.uk/) under application number 55469. Research Training Program (RTP) Stipend PhD scholarship from Monash University (2020) and the Alice Baker and Eleanor Shaw Gender Equity Fellowship, Baker Trustees.

19	**Data and data sharing**	Provide the data used to perform all analyses or report where and how the data can be accessed, and reference these sources in the article. Provide the statistical code needed to reproduce the results in the article, or report whether the code is publicly accessible and if so, where	21	The sleep GWAS summary data were obtained from the Sleep Disorder Knowledge Portal (http://sleepdisordergenetics.org/) and the BP GWAS summary data were obtained from the UK Biobank cohort and International Consortium of BP Genome-Wide Association Studies (ICBP).29
20	**Conflicts of Interest**	All authors should declare all potential conflicts of interest	29	No conflicts of interests are declared.

This checklist is copyrighted by the Equator Network under the Creative Commons Attribution 3.0 Unported (CC BY 3.0) license.

1. Skrivankova VW, Richmond RC, Woolf BAR, Yarmolinsky J, Davies NM, Swanson SA, et al. Strengthening the Reporting of Observational Studies in Epidemiology using Mendelian Randomization (STROBE-MR) Statement. JAMA. 2021;under review.

2. Skrivankova VW, Richmond RC, Woolf BAR, Davies NM, Swanson SA, VanderWeele TJ, et al. Strengthening the Reporting of Observational Studies in Epidemiology using Mendelian Randomisation (STROBE-MR): Explanation and Elaboration. BMJ. 2021;375:n2233.
